# Thin‐Wall Single‐Crystal Gold Nanoelectrodes toward Advanced Chemical Probing and Imaging

**DOI:** 10.1002/smll.202514938

**Published:** 2026-02-26

**Authors:** Milad Sabzehparvar, Fatemeh Kiani, Germán García Martínez, Omer Can Karaman, Victor Boureau, Lucie Navratilova, Giulia Tagliabue

**Affiliations:** ^1^ Laboratory of Nanoscience for Energy Technologies (LNET) STI École Polytechnique Fédérale de Lausanne Lausanne Switzerland; ^2^ Interdisciplinary Center for Electron Microscopy (CIME) École Polytechnique Fédérale de Lausanne Lausanne Switzerland

**Keywords:** biosensor, crystal growth, electrochemical microscopy, electrochemical sensor, high‐throughput fabrication, metal nanoelectrodes, near‐field microscopy, single crystal

## Abstract

Thin‐wall metal ultramicro‐ and nanoelectrodes (UMEs/NEs), especially gold‐based ones, are key probes for high‐resolution electrochemical microscopy, biosensing, and nanoscale interfacial studies. Yet, their broader use remains limited by fragility, low detection sensitivity, and the lack of scalable fabrication methods. Here, we present a template‐assisted, non‐self‐limited polyol‐based growth strategy that realizes single‐crystalline, thin‐walled Au UMEs/NEs, as well as multifunctional probes, with high yield (>80%). The method provides precise control over electrode dimensions, from sub‐100 nm to micron‐scale radii while the massively parallel polyol growth step overcomes the key bottleneck in scalable nanoelectrode preparation by reliably producing long, continuous single‐crystal metal cores. Structural and electrochemical characterization confirm twinned single‐crystal Au cores, seamless Au/glass interfaces, and stable performance. Through direct comparison across radii, we show that smaller electrodes consistently exhibit higher surface reactivities, boosting chemical detection sensitivity. In scanning photoelectrochemical microscopy, these NEs achieve an illumination‐dependent spatial resolution of ∼250 nm, <1 pA current sensitivity, a detection limit of ∼11.0 µm, and over 7 h of operational stability. In bulk electrolytes, the single‐crystalline electrodes achieve ultralow detection limits down to 79 nm, markedly enhancing the signal‐to‐noise ratio in nanoscale electrochemical measurements. Finally, we demonstrate growth in double‐barrel pipettes for multifunctional probes and extend the approach to Pt NEs. This scalable method overcomes longstanding limitations in NE fabrication, enabling advanced electrochemical imaging and its combination with tip‐enhanced spectroscopic methods. The single‐crystalline architecture also opens new frontiers in catalysis, interfacial electrochemistry, biosensing, and molecular‐scale investigations.

## Graphical abstract

We present a template‐assisted, non‐self‐limited polyol‐based growth strategy that realizes single‐crystalline, thin‐walled Au UMEs/NEs, as well as multifunctional probes, with high yield (>80%). The method provides precise control over electrode dimensions, from sub‐100 nm to micron‐scale radii and reliably produces long, continuous single‐crystal metal cores. Enabling physical contacting this method enables high‐performance nanoelectrode probes for advanced electrochemical imaging.

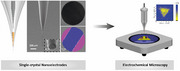

## 1 Introduction

**FIGURE 1:**
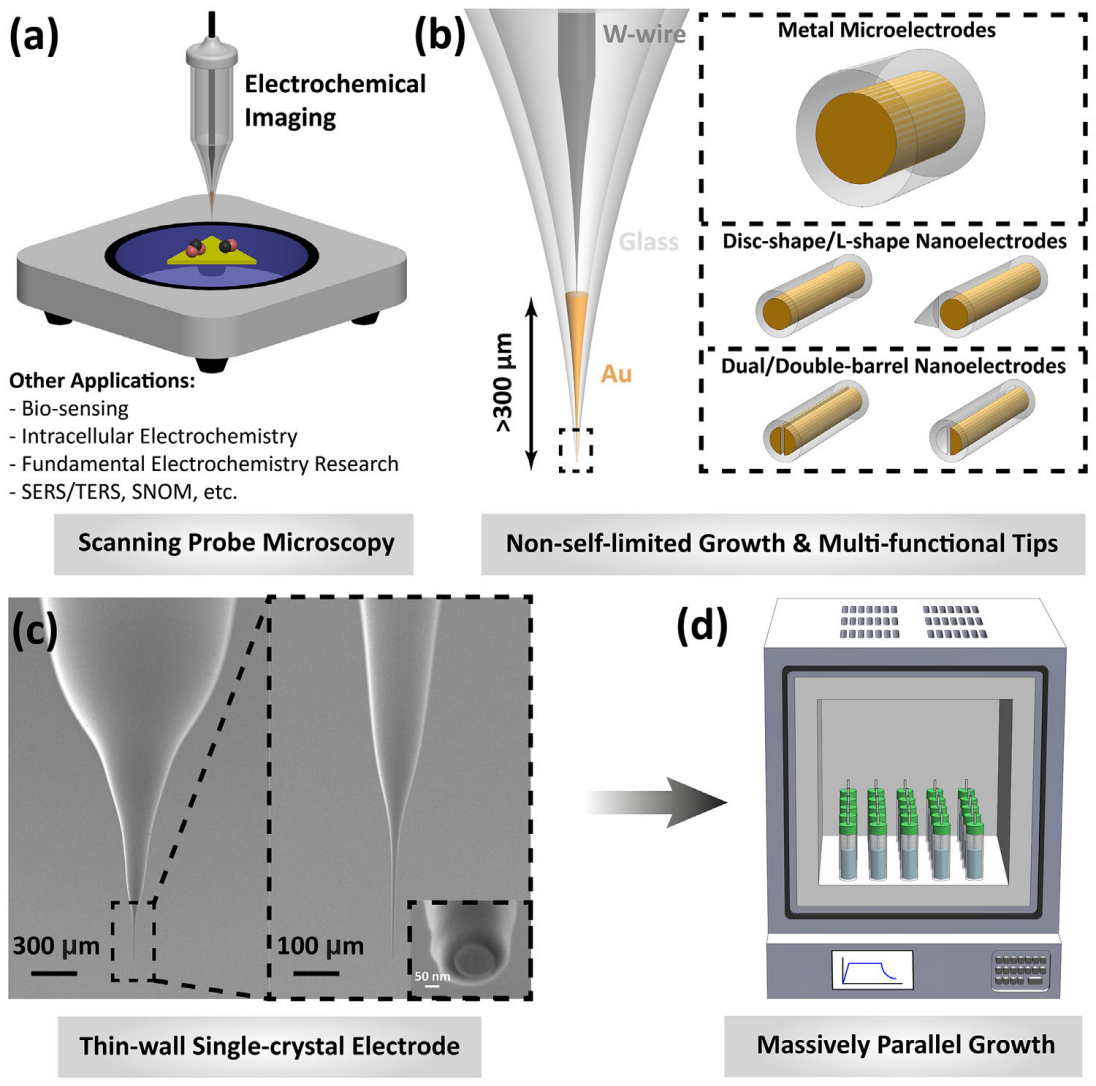
Thin‐wall metal nanoelectrodes. (a) Schematic illustration of the implementation of the electrodes for electrochemical microscopy technologies (e.g. SECM, EC‐STM, and SECM‐AFM), and other potential applications. (b) Schematic illustration demonstrating flexibility of the wet‐chemical approach in obtaining various physically‐contacted thin‐wall electrochemical probes having different material, geometry, and sizes (see experimental examples in Figure 4c–f). Using salt of different metals can enable fabrication of different metal NEs. Complete chemical growth of gold metal inside single‐barrel and asymmetric double‐barrel nanopipettes enables high‐throughput fabrication of disc‐shape gold micro‐/nanoelectrodes and multi‐functional nanoelectrodes. A partial cross‐sectional FIB cutting enables fabrication of recessed L‐shape metal nanoelectrodes. (c) SEM images of a typical thin‐wall gold nanoelectrode with a disc‐shape geometry. (d) Schematic illustration demonstrating the mass production of metal nanoelectrodes using a wet‐chemical growth approach inside a muffle furnace. Multiple glass vials are processed simultaneously within the furnace, showcasing the scalability of the fabrication method for large‐scale production of nanoelectrodes.

Nanoelectrodes (NEs), with radii below ∼100 nm, serve as powerful probes for investigating electrochemical processes at the nanoscale [1, 2]. This is largely due to their size being much smaller than the diffusion layer thickness, resulting in extremely high mass transport rates, low ohmic losses, small RC time constants, and fast steady‐state responses [1, 3]. These unique advantages enable investigation of numerous phenomena and processes that cannot be studied at macroscale electrodes, including electrochemistry of individual molecules [4, 5, 6] and nanoparticles [7, 8, 9, 10], formation and growth of transient metal nuclei [2, 11], nanobubbles [13, 14, 15], short‐lifetime intermediates [16, 17], rapid heterogeneous electron transfer kinetics [18, 19, 20], and non‐invasive electrochemistry inside living cells [21]. Among all metals, gold NEs find more extensive applications in bio‐analysis [22, 23, 24] and catalysis [25] research due to their inertness, high surface reactivity and higher affinity for biomolecules and chemical bonds (Figure 1a).

Laser pulling of glass‐sealed metal microwires is a common technique for fabricating disc‐shape metal NEs [26, 27]. However, despite the huge improvements in the past decades for achieving platinum ultramicro‐/nanoelectrodes (UMEs/NEs) [26, 28], challenges still exist to reproducibly and massively produce high‐quality, “leak‐free”, gold NEs. This is primarily due to the large difference between the melting point of gold (1064°C) and quartz (1710°C), resulting in low success rate for pulling continuous gold NEs and a poor seal at the gold/glass interface. This leads to an unstable electrochemical performance [18] and necessitates transmission electron microscopy (TEM) for controlling the fabrication [29], severely limiting the throughput of the process. In addition, laser‐pulled electrodes, while exhibiting some degree of grain alignment, are usually polycrystalline with randomly oriented crystallites and grain boundaries [30, 31]. Most importantly, the resulting NEs typically feature a thick glass insulation sheath, with a glass‐to‐conductive core radius ratio (known as the RG value) substantially exceeding 10, resulting in overall dimensions within the micrometer scale [27].

Nanoelectrodes with small RGs, e.g. 1.2–2, have emerged as critical tools for probing neurotransmitters, and achieving ultrahigh spatial resolution in chemical [32, 33] and spectroscopic (near‐field) imaging [34, 35, 36, 37]. This originates from their small footprint, minimizing the mechanical damage to a cell in neurobiology and allowing extremely small tip‐to‐substrate distances in scanning electrochemical microscopy (SECM), critical for enhanced detection sensitivity [32]. Despite huge efforts in advancing the fabrication of such NEs, for example by using a thin polymer coating on metal micro‐/nanowires [38], or by reducing the thickness of the glass sheath of laser‐pulled NEs by chemical etching [39], mechanical polishing [40], or local heating [33, 41], these processes remain either very time‐consuming and challenging or frequently result in a defective insulating sheath having parasitic current leakage.

Glass nanopipettes, instead, are thin‐wall nano‐pore structures that are nowadays easily fabricated with sub‐50 nm orifice sizes at uniquely high yield and repeatability [42, 43, 44]. Recently, room‐temperature template‐assisted chemical and electrochemical deposition of metals in glass nanopipettes has been found as a promising approach for controlled fabrication of low‐RG‐value metal NEs [45, 46, 47, 48]. However, crystal growth is a self‐limited process in all these methods, resulting in short‐length deposits and limiting the performance only to a wireless bipolar electrochemical contacting approach [49, 50]. Moreover, the yield of these serial fabrication processes remains low, and poor sealing at metal/glass interface, especially for larger‐size NEs [48], remains an issue, leading to a high background ionic current in addition to the Faradaic current detection. Thus, it is necessary to develop a high‐throughput processing method for deposition of very long length Au crystals and realization of high‐quality physically‐contacted thin‐wall Au NEs.

In this work, we present a polyol‐based method for template‐assisted 1D growth of single‐crystalline gold within borosilicate nanopipettes, overcoming the longstanding bottleneck of scalable fabrication of physically contacted, thin‐walled gold NEs (Figure 1). Leveraging heated ethylene glycol as both solvent and weak reducing agent, our process uniquely enables non‐self‐limited crystal growth, yielding continuous single‐crystalline gold deposits extending over hundreds of microns and allowing reliable physical contacting (Figure 1b). While serial template preparation and tip finishing steps remain, the massively parallel polyol growth step eliminates the key barrier to producing continuous single‐crystal metal cores at scale (>80% batch success rate; Figure 1c,d), unlike previous wet‐chemical approaches [45, 46, 47, 48]. The resulting electrodes uniquely combine low RG values (down to ∼1.6), tunable radii from ∼50 nm to >2 µm, seamless gold–glass interfaces, and robust long‐term stability in both aqueous environments and air. Leveraging the precision of glass capillary pulling, the method is inherently versatile (Figure 1b), supporting multifunctional designs (dual, double‐barrel, recessed L‐shape) and is extendable to other metals of technological importance (Pt, Ag, Cu, Pd, Bi). Beyond fabrication, we uncover a size‐dependent reactivity during hydrogen evolution reaction, where smaller single‐crystalline nanoelectrodes display enhanced activity and sensitivity, achieving bulk detection limits as low as 94 nm (79 nm for microelectrodes). We further demonstrate their viability for photo‐SECM by imaging weak photo‐oxidation over atomically flat gold microflakes with subwavelength spatial resolution (∼250 nm, illumination dependent), ultralow current step detection (<1 pA), excellent detection limits (∼11.0 µm), and long‐term operational stability (>7 h). Altogether, this approach establishes a high‐throughput and generalizable route to single‐crystalline metallic nanoelectrodes, overcoming the challenge of reliably producing long and continuous metal cores. The combination of single‐crystalline gold and thin‐walled geometry also unlocks new opportunities for high‐performance nanoelectrochemistry, including integration with near‐field spectroscopies [51, 52] (e.g., SERS/TERS), toward probing fundamental interfacial processes such as double‐layer structure and biomolecular interactions [15, 53].

## 2 Results

### 2.1 Gold Nanoelectrode Fabrication

**FIGURE 2:**
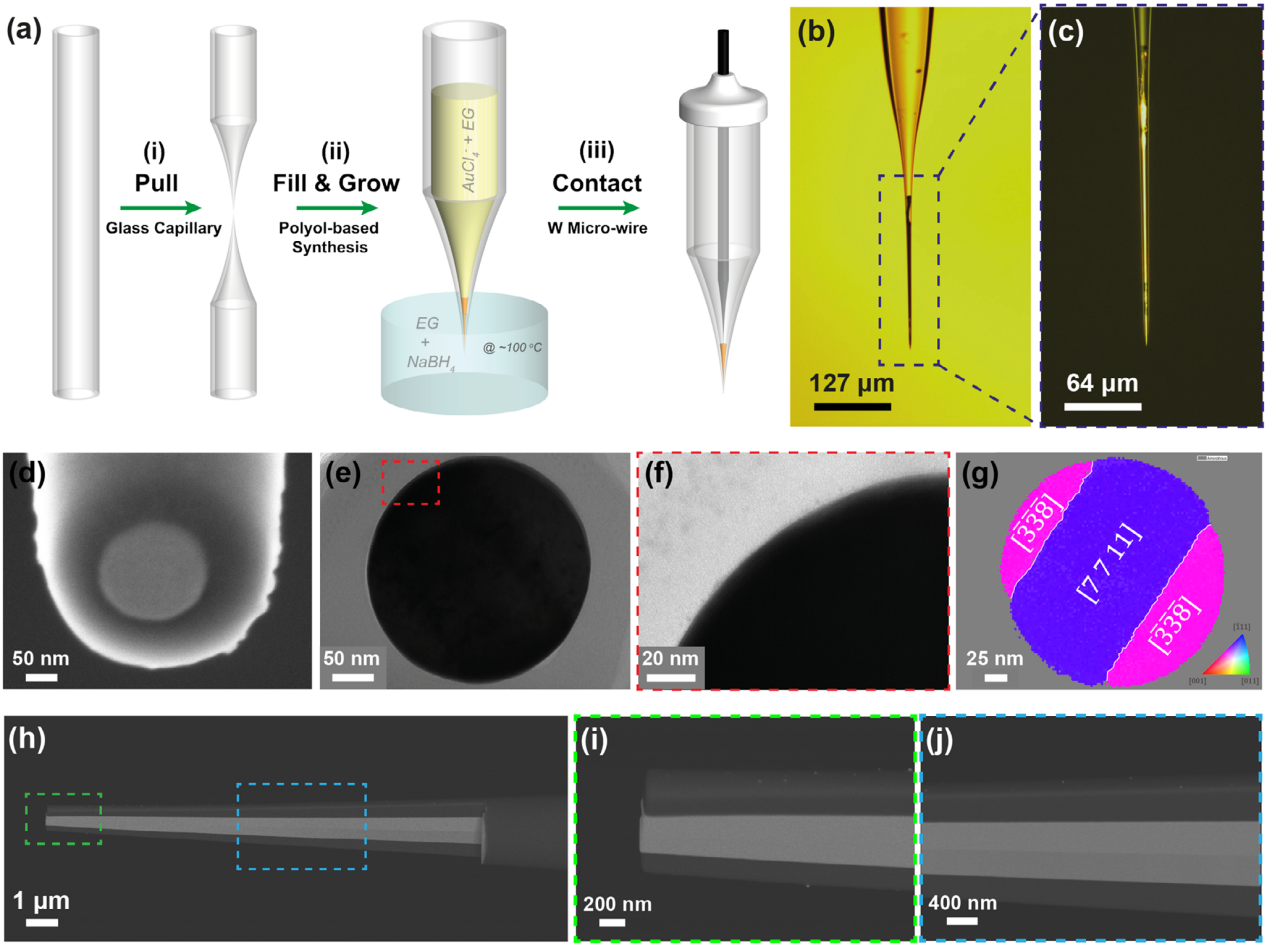
Fabrication process and quality analysis for thin‐wall single‐crystalline gold nanoelectrodes. (a) Schematic illustration of wet chemical growth of thin‐wall gold nanoelectrodes. (i) Pulling of glass capillaries into sub‐20 nm glass nanopipettes. (ii) Filling the nanopipettes with a growth solution containing AuCl_4_^−^ ions in ethylene glycol and insertion of the filled nanopipette into a bulk media containing ethylene glycol and sodium borohydride. Huge gold crystal grows inside the nanopipette via a polyol‐assisted process. (iii) Physical contacting by connecting an electrochemically sharpened tungsten micro‐wire to the large‐size gold deposit. Transmission (b) and reflection‐mode (c) optical micrographs of an as‐grown >200 µm long, gold deposit inside the glass nanopipette. (d) Tilted‐view SEM image of a FIB‐cut gold nanoelectrode at 54˚ angle. (e) Top‐view BF‐TEM image of a sliced lamella of gold nanoelectrode. (f) High‐magnification BF‐TEM image of the gold‐glass interface marked with a red dashed rectangle in (e). (g) Orientation map of the Au crystal with respect to the axis of the nanoelectrode shown in (e), with {111} Σ3 twins overlayed in white. The inset shows the corresponding color legend. (h–j) SEM images of an axially FIB cut Au NE measured by an in‐lens backscattered electron detector. They show the perfect sealing and single‐crystallinity of the gold deposit along the glass capillary. The Au NE was exceptionally coated with a 10 nm carbon layer for decreasing the charging and drifting problem during the FIB cutting process.

A thin‐wall (i.e. low‐RG‐value) NE is defined as an electrode with an RG value smaller than 10, preferably less than ∼2, which corresponds to a wall thickness equal to the core radius and an optimum back‐diffusion of chemical species (Section S1). Fabrication of thin‐wall Au NEs was achieved by developing a unique polyol‐based wet chemical growth process inside glass nanopipette templates, inspired by a 2D growth method for single‐crystalline Au micro‐flakes on glass substrates that we recently reported [54]. The process involves three main stages as illustrated in Figure 2a. Briefly, borosilicate glass nanopipettes are fabricated by a laser pulling process (Figure 2a.i) and used as templates for selective growth of long‐length gold deposits at the very end of the nanopipette (Figure 2a.ii,b,c) followed by its physical contacting to an electrochemically sharpened tungsten micro‐wire under an optical microscope (Figure 2a.iii). A tip finishing step, like focused ion‐beam milling (FIB) or gentle mechanical polishing [55], is finally performed to cut any protruded Au deposit and realize perfect disc‐shape Au NEs. We note that our growth method can result in NEs with a nanosized conical protrusion (Figures S5b and S10d), or even disc‐shapes (Figure S6), excluding the need for the final step for applications, e.g. biosensing [23] and spectroscopic [37, 56] imaging, where roughened surfaces are preferred over ideal disc geometries [23, 57, 58].

Glass nanopipettes with exceptionally short‐taper (∼300 µm) and ultra‐fine (sub 20 nm) openings are fabricated by an optimized combination of laser‐shrinking and laser pulling of borosilicate glass capillaries having large inner diameters (Section S2 and Methods for complete details). Critically, the excellent (∼90%) reproducibility of such ideal‐shape borosilicate nanopipettes (Figures S2b and S3b), ensures the overall reliability of the nanoelectrode fabrication method.

For fabrication of Au NEs (Section S3 and Methods), the glass nanopipettes are back filled with a growth solution containing AuCl_4_^−^ ions in ethylene glycol (EG) using a home‐built glass injector, and then immediately inserted vertically in glass vials containing EG and sodium borohydride (NaBH_4_). Next, the vials are promptly transferred to a furnace heated at 110˚C for accelerating the gold deposit growth via a polyol synthesis process. During the heating process, the aldehyde groups (─CHO) of the heated EG continuously reduce the AuCl_4_^−^ ions into gold nanocrystals [54] whose directional growth is then accelerated by the strongly reducing BH_4_^−^ ions. The temperature‐dependent aldehyde formation in EG is key in suppressing unwanted nanoparticle deposition and enabling selective growth of large gold deposits even after the nanopipette orifice has closed, thereby overcoming the self‐limited internal growth typical of conventional wet‐chemical methods [45, 46, 47].

We performed several concentration studies revealing that (i) gold nucleation is initiated by a confinement effect close to the orifice rather than randomly across the nanopipette body (Figure S8), (ii) diffusion of both NaBH_4_ and AuCl_4_^−^ is critical for the growth process, the latter determining the morphology of the growing crystal (Figures S8 and S9), and (iii) a time‐dependent concentration (increasing) of AuCl_4_^−^ enables selective growth of long gold deposits while minimizing unwanted nanoparticle on the outer surface of the glass capillary (Figure S10b,d). To achieve the latter, the nanopipette tip is thus first dipped in pure EG while a high AuCl_4_^−^ solution is backfilled. We also observed that optimized growth conditions are necessary to avoid a severe chemical attack to the thin glass close to the nanopipette apex at both high AuCl_4_^−^ ions and NaBH_4_ concentrations, an effect that is strongly enhanced by increasing the growth temperature and time (Figures S8,S9, and S11). After a final FIB‐milling step, which allows straightforward inspection and controlled removal of any damaged glass section, we reach a disc‐shaped gold electrode (Figure 2d; Figure S5c,d). Overall, we found that 0.125 m AuCl_4_^−^ in EG growth solution, 0.5 mL of 200 mm NaBH_4_ in ethanol in 2 ml EG bulk media, 110˚C growth temperature and 24 h growth time are optimized conditions for obtaining small‐size Au NEs. When using ∼20 nm radius glass nanopipettes as starting point, this process results in ∼80% success rate for achieving around 300 µm continuous gold deposits completely filling the conical part of the nanopipette with the least amount of unwanted growth residues in the pipette stem and on the exterior surfaces (Figure 2b,c; Figure S4). After FIB cutting, a sub‐100 nm‐radius Au NE can be reliably obtained (Figure 2d; Figure S5c).

The ∼300 µm typical gold deposit length obtained with the described method is about 130, 10, and 15 times higher than the ones previous obtained by bipolar electrochemistry [47], electrochemical deposition [46] and interfacial reaction [45] methods, respectively. Together with the optimized nanopipette short‐taper, it ultimately enables standard physical contacting of the NEs using metal wires. This is indeed critical to overcome unavoidable limitations of bipolar electrochemical contacting such as poor long‐term stability and current‐concentration nonlinearity [49, 50, 59] (Section S5). Reliable electrical connection was achieved with >90% reproducibility, either by a simple tungsten micro‐wire contacting strategy (Section S6 and Figure S15) or directly using commercially available 25 µm W wires for exceptionally long Au deposits (Figure S17). Overall, although serial template preparation and tip finishing steps remain unavoidable, the massively parallel polyol growth step (Figure 1d; Figure S4) allows to reliably obtain continuous single‐crystal metal cores at scale, eliminating a key barrier to massively producing tens of nanoelectrodes for advanced electrochemical probing and imaging applications.

### 2.2 Material Characterization

Quality of the fabricated electrodes was assessed by electron microscopy and via an electrochemical analysis approach, to resolve details of the physical and chemical properties, respectively. Figure 2d shows an SEM image of a typical Au NE after physical contacting and FIB cutting processes. The Au disc is highly circular with a geometrical radius of 85 nm, the glass sheath is thin (∼85 nm) and the gold/glass interface is free of visible (>nm‐scale) gaps. Axial FIB cutting on a different Au NE showed that the gold/glass interface remains equally seamless even up to tens of micrometers from the very end (Figure 2h–j). This suggests a complete chemical growth of Au, filling its template shape, and promises a large flexibility for the fabrication of Au NEs having different sizes by this approach (Figure S5c,d; Figure 4a,b; Figure S28a–e), from microelectrodes to nanoelectrodes. Bright‐field (BF) TEM imaging of a thin FIB cross‐section of the electrode shown in Figure 2d further confirmed the seamless quality of gold/glass interface (no gaps, Figure 2e,f), indicating a complete chemical growth for the Au deposit.

Remarkably, TEM images revealed the single crystalline nature of the gold deposit. A precession‐assisted TEM experiment for crystal orientation mapping [60] (Figure 2g) showed the existence of characteristic twins in gold, precisely Σ3 twin boundaries, characterized by a 60° rotation around <111> crystal axis (Figure S19f) [61]. The twinning planes are parallel to {111} planes for crystal grains at both sides, revealing coherent twins, precisely {111} Σ3 grain boundaries (Figure S20a,c). Crystal domains are oriented [7 7 11] and [−3,−3,−8] along the NE axis, for the bigger and the two smaller domains, respectively. The orientation mapping on a bigger sized NE (Figure S19a–c) prepared by FIB cross‐sectioning further from the tip showed the same {111} Σ3 twinned boundary structure with increased number of crystal grains as well as [7 5 11] and [−6,−4,−11] orientations along the NE axis, for the bigger and for the smaller domains, respectively. These orientations reveal a preferred close‐to‐<111> orientation, as highlighted by the plot of the inverse pole figures along the NE axis where the intensities are distributed closer to the [111] pole (Figure S20b,d). The misorientation of the <111> crystal axis of the larger grain with respect to the NE axis is 18.0˚ for the bigger NE, but only 12.7˚ for the smaller NE. The authors anticipate that smaller nanoelectrodes (<100 nm radius) would exhibit crystallographic orientations closer to <111>. Some clues to understand this phenomenon can be related to a slight change in the crystal growth trajectory during the growth process inside the conical nanopipette channel observed at micrometer scale (Figure S22), or the change in position of the twin boundaries inside the Au disc observed at nanometer scale with serial FIB cross‐sectioning (Figure S21) both resulting from the misalignment between the <111> crystal axis and the pipette axis. Further in‐depth studies are needed to investigate the underlying growth mechanism as well as its role in the formation of the record‐large metal deposit achieved through our polyol‐based method.

### 2.3 Electrochemical Characterization

**FIGURE 3:**
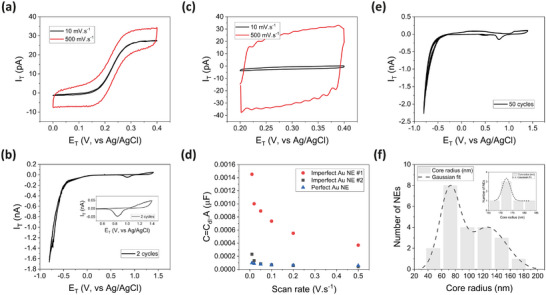
Electrochemical characterization, electrode stability and process reproducibility. (a) Cyclic voltammograms of a nanoelectrode analogous to Figure 2d in an electrolyte solution containing 1 mm ferrocene methanol (FcMeOH) redox molecules in 0.125 m KCl_(aq.)_ at low and high scan speeds. A faraday cage was used for these measurements. (b) Two‐cycles cyclic voltammograms of the nanoelectrode, from HER to gold redox potential windows, at 10 mV.s^−1^ in a 50 mm H_2_SO_4_ electrolyte. The inset shows perfect overlap of the gold redox peaks over two consecutive cycles of CVs up to the HER triggering regime, i.e. −0.8 V vs Ag/AgCl. (c) Cyclic voltammograms of the nanoelectrode in the same electrolyte as (b) at 10 and 500 mV.s^−1^ scan speeds within the double‐layer potential window. (d) Plot of total capacitance as a function of scan rate for the perfect Au NE in Figure 2d and imperfect NEs in Figure S27a,d. (e) Stability of a ∼80 nm‐radius Au NE over 50 cycles (∼70 min) at 50 mV.s^−1^ in a 50 mm H_2_SO_4_ electrolyte. (f) Statistical analysis results for disc‐shape nanoelectrodes fabricated in the same batch (total of 26 empty nanopipettes) by the wet chemical approach. The inset shows analysis result for disc‐shape Au NEs obtained by controlled FIB cutting for a specific electrode radius, e.g. ∼170 nm.

To test the electrical contact of the NEs and electrochemically examine their geometrical size we performed outer‐sphere cyclic voltammetry experiments in redox couple solutions [62] (see Methods). Figure 3a shows the voltammetric response of a Au NE analogous to Figure 2d at two different scan rates in an electrolyte solution containing 1 mm ferrocene methanol (FcMeOH) in 0.125 m KCl. It can be seen that the electrode exhibits a well‐defined sigmoidal CV with a plateau diffusion limiting current and almost no hysteresis at low scan rates, i.e. 10 mV.s^−1^. Importantly, it remains in this steady‐state condition even at scan rates as high as 0.5 V.s^−1^. This indicates the good electrical contact and high surface reactivity of the electrodes, and confirms the absence of any solution‐filled gap at the gold/glass interface [18, 63], as well as an inner‐sphere interaction between gold and ferrocene [64]. We note that measurements on Au NEs/UMEs in a 2 mm ferrocyanide solution in 0.25 m Na_2_SO_4_ instead exhibited a dependence on the oxidative potential window: scanning to higher oxidative potentials led to a flatter mass‐transport‐limited regime accompanied by increased hysteresis (Figure S23). This behavior arises from an inner‐sphere oxidative dissolution process between gold and cyanide [59, 65], occurring concurrently with the outer‐sphere electro‐oxidation of Fe(CN)_6_^4−^. In fact, when tested in a FcMeOH electrolyte—governed by a purely outer‐sphere mechanism on gold—the same electrodes displayed textbook voltammograms with a flat mass‐transport‐limited regime at low potentials and minimal hysteresis, which remained negligible even at scan rates as high as 2 V·s^−^^1^ (Figures S23c,d and S24) [66]. We therefore caution against using gold nanoelectrodes for ferro‐/ferricyanide studies, as the unwanted interactions compromise stability and reliability of the electrodes.

Considering a disc‐shape NE with thin glass walls, the radius of the electrode can be calculated from the diffusion‐limited steady‐state current:

(1) iT,∞=gnFDC∗r

where n is the number of electrons transferred, F is Faraday's constant, r is the core radius, D and C^*^ are the diffusivity and bulk concentration of Fe(CN)_6_^4−^ molecules, respectively. The geometrical factor g is dependent on the glass wall thickness, as follows,

(2) $$g=4.00+4B{\left(RG-C\right)}^{D}$$

Using the literature‐established values of B = 0.1380, C = 0.6723, D = −0.8686, [67, 68] and an RG value of 2 for the electrode, the measured 26 pA limiting current corresponds to an estimated electrode radius of 90 nm that closely matches the geometrical radius estimated by SEM (∼85 nm).

Since outer‐sphere voltammetry is nonetheless largely insensitive to surface composition and nanoscale imperfections [69], both perfect and imperfect Au NE/UMEs can exhibit excellent CV responses (Figure S23). Thus, blank voltammetry in H_2_SO_4_ has been established as a more reliable approach for evaluating surface chemistry, electrochemical surface area (ECSA), and electrode roughness (RF value) [62]. Figure 3b shows two consecutive CV cycles of the same NE in a 50 mm H_2_SO_4_ solution at 10 mV.s^−1^ scan rate in a potential window covering both the hydrogen evolution reaction (HER) and gold redox processes. The presence of clear gold oxidation and reduction peaks at 1.4 and 0.84 V vs Ag/AgCl is indicative of a clean gold surface [18, 62]. From the consumed charge under the gold reduction peak (390 µC.cm^−2^), we estimated a RF value of ∼7, in line with those of typical NEs (between 1.2 and 12), due to likely deviations from a 1:1 stoichiometric monolayer formation of gold oxide/hydroxyl and its reduction at nanoscale [70, 71, 72]. Our CV measurements at varying oxidative potentials clearly show that the integrated reduction charge increases with the maximum potential applied [73], resulting in different apparent ECSAs and consequently in over‐ or underestimation of electrode size if the measurements are not confined to a potential window corresponding to 1:1 stoichiometric monolayer formation. On the other hand, the sealing quality (gold/glass interface quality) of the NEs, can be reliably confirmed by two features of the CV scans: (i) the highly retraceable CV curves in the HER and gold redox potential regimes (Figure 3b), as any interfacial defect would lead to unreproducible measurements, and (ii) the flat profile of the CV baseline within the double layer regime up to scan rates as high as 0.5 V.s^−1^ (Figure 3c), as defect would otherwise result in tilted scans due to resistive contributions [69, 74]. Additionally, the negligible (<2 times) dependency of the calculated double‐layer capacitance (C_dl_) on the scan rate (Figure 3d), especially at scan rates smaller than 100 mV.s^−1^, further supports the gap‐free nature of the gold/glass electrochemical interface, as defects could result in orders of magnitude increase in the C_dl_ value due to the variation of the iR drop [74]. Indeed, experiments on selected defective NEs having different gap sizes confirm for the first time the unique sensitivity of these assessment techniques to sealing quality (Figure 3d; Figures S26 and S27), —beyond the reach of conventional outer‐sphere voltammetry (Figure S26).

### 2.4 Gold Nanoelectrode Stability

Stability of the electrodes was also evaluated by performing 50 cycles of CVs within the HER and gold redox regimes, corresponding to ∼75 min of electrochemical experiment in 50 mm H_2_SO_4_ solution. As can be seen from Figure 3e, showing all 50 cycles, there is no obvious change in the CV response of the nanoelectrode, supporting the sealing quality inferred from the electrochemical analyses. Consistently, repeated outer‐sphere CV cycling in FcMeOH electrolytes demonstrated excellent stability for our gold electrodes (Figure S26a,b), whereas inner‐sphere cycling again revealed clear differences between perfect and imperfect electrodes (Figure S26c,d). Nonetheless, SEM imagining before and after the cycling treatments shows a slight recession for the NE due to electrochemical etching of gold during its redox process (Figure S31d,e). While this is an inevitable damaging mechanism for small NEs [41], in our tips it interestingly happens in a smooth, layer‐by‐layer manner due to the single‐crystalline nature of the grains, and the low‐energy coherent nature of the {111} Σ3 twin boundaries in our Au NEs [61]. Thus, the twin boundaries are still evident on the tested electrodes and, most importantly, the gold/glass interface does not deteriorate. This allows prolonged operation and uniquely enables the possibility of easy re‐using the electrodes after a recovery FIB cutting process (Figure S31f) or a gentle mechanical polishing. Notably, the electrodes exhibited nearly identical electrochemical performance even after long‐term storage (e.g., over three months) in air, demonstrating exceptional stability in electrical connection and surface reactivity (Figure S31c).

Overall, our comprehensive physical and electrochemical characterizations indicate the high quality of the single‐crystalline Au NEs fabricated via the polyol‐based growth method, ensuring a stable electrochemical performance for advanced scientific research. This represents a significant breakthrough in the fabrication of high‐quality thin‐wall Au NEs, overcoming longstanding challenges. Unlike secondary electrochemical deposition methods using recessed NEs, which often result in porous Au structures with inconsistent and unpredictable electrochemical behavior [23, 75], our approach reliably delivers solid and gap‐free thin‐wall disc‐shape electrodes with excellent electrochemical performance and durability.

### 2.5 Reproducibility and Size Distribution

To quantify the reproducibility, reliability, and scalability of our overall fabrication protocol, we measured the size distribution of one batch of fabricated Au NEs from their SEM images (Figure 3f). In this instance, we pulled 13 glass capillaries into 26 identical nanopipettes having a ∼20 nm‐radius orifice size (>90% yield). Importantly, our polyol‐based growth step enables massively parallel preparation of long and continuous Au cores inside these templates, which has not been achieved by prior wet‐chemical growth methods. Conventional laser pulling of glass‐sealed metal wires is a serial process and often results in discontinuous cores and poor reproducibility, whereas our approach allows the simultaneous production of tens of gold‐filled nanopipettes with high reliability. After the parallel chemical growth, we performed serial physical contacting and FIB cutting processes, obtaining 22 Au NEs with large‐size Au deposits and perfect disc‐shape geometry (>80% reproducibility). The discarded NEs either had a relatively shorter Au deposit or a slight gap at the gold/glass interface. Remarkably, the size distribution is very narrow, spanning from ∼55 to ∼170 nm. Importantly, the as‐grown size distribution can be further narrowed by controlling the cutting size during the FIB step toward a specific electrode size, e.g. 175 ± 10 nm (see inset in Figure 3f; Figure S7b–d). Overall, by benefiting from the highly reliable glass capillary pulling process and, most critically, from the massive parallelization of the growth step, the reported approach offers high‐throughput and controllable fabrication of metallic nanoelectrodes with a specific size — a capability that has not been demonstrated before in the metallic nanoelectrode literature and that is essential for many applications.

### 2.6 Size Effects on Electrochemical Response

An interesting possibility opened by our approach, is the direct comparison of the electrochemical performance of Au NE with different radii. In fact, the size of the Au NE depends on the nanopipette template and the single crystalline nature of the Au offers excellent control onto the tip material. We thus repeated our detailed quality assessment on disc‐shape Au NEs having sizes ranging from ∼45 to ∼550 nm radii. Figure S28a–e shows SEM images of the studied electrodes. From the outer‐sphere (Fe(CN)_6_^4−^ oxidation) voltammograms (Figure S30a), we confirmed that the electrochemically calculated radius is similar to the geometrical values measured from SEM images. As noted before by purely outer‐sphere FcMeOH testing (Figures S23 and S24), any observed hysteresis, cross‐over, or deviations from a flat mass‐transport‐limited regime arise from inner‐sphere interactions between gold with Fe(CN)_6_^4−/3−^ [59, 64, 65], rather than from electrode imperfections. Normalization of the CVs to the limiting current values of the oxidation waves revealed no obvious size‐dependency for the half‐wave potential, E_1/2_, and thus surface reactivity for Fe(CN)_6_^4−^ oxidation on different size electrodes (Figure S30b). This is in agreement with the surface insensitivity of this fast reaction based on outer‐sphere charge transport mechanisms [64, 69].

**FIGURE 4:**
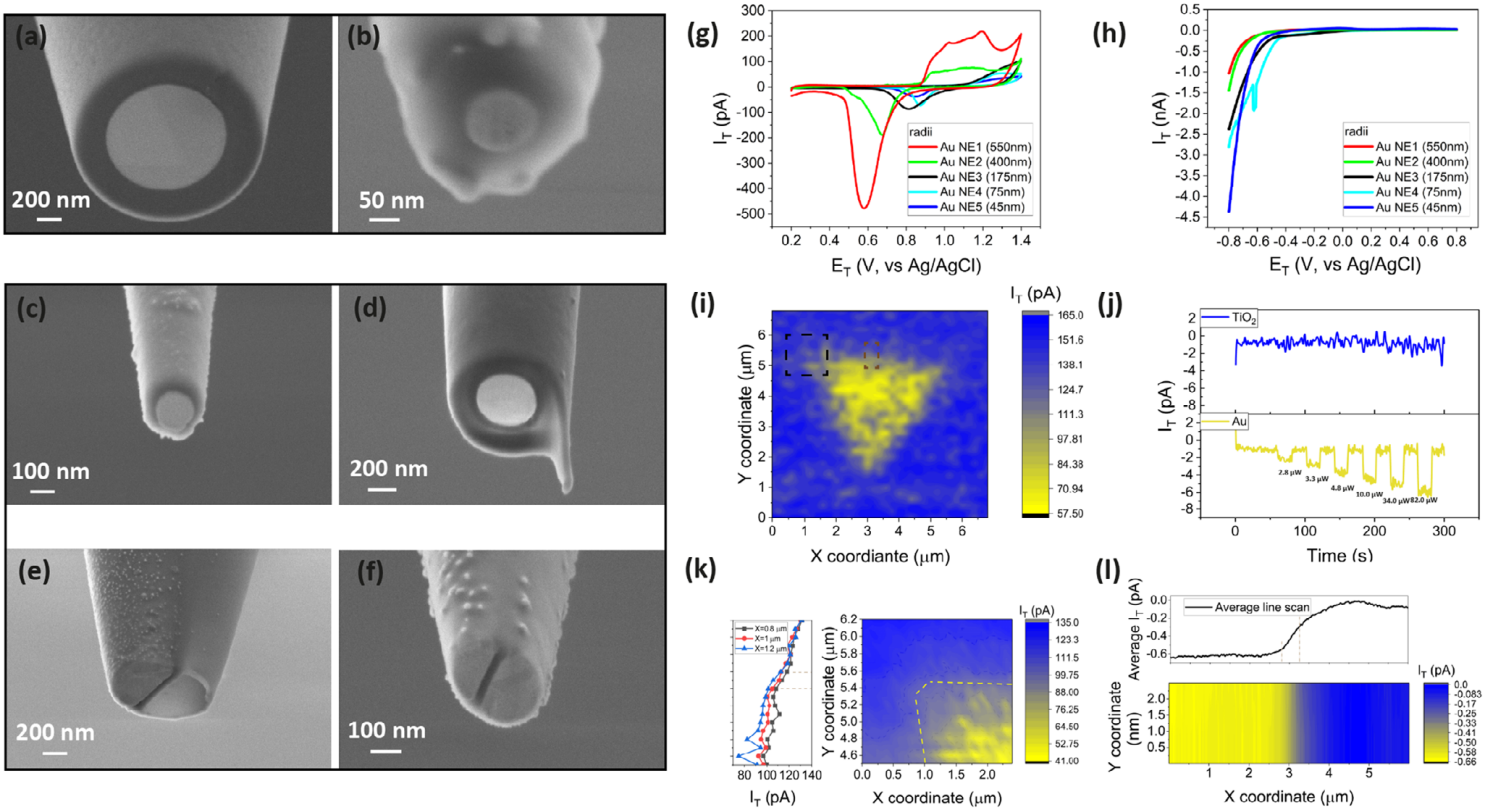
Electrode versatility, size effect, and photoelectrochemical imaging. (a,b) Tilted‐view SEM images of a disc‐shape Au NE and an Au UME having different core sizes (Figure S28a–e for all electrode sizes). (c–f) Tilted‐view SEM images of (c) a platinum nanoelectrode, (d) a recessed L‐shape gold nanoelectrode, (e) a double‐barrel gold nanoelectrode (left barrel: Au, right barrel: empty), and (f) a dual gold nanoelectrode. (g) Cyclic voltammograms of the gold nanoelectrodes in 50 mm H_2_SO_4_ within the gold redox and (h) HER regimes. (i) Constant‐height photo‐SECM image of photo‐oxidation of Fe(CN)_6_^4−^ molecules on a single‐crystalline gold micro‐flake surface on a TiO_2_ substrate, analogous to Figure S32b. The image was obtained with a 200 nm‐radius Au NE biased at a 0.4 V vs Ag/AgCl in a 4 mM Fe(CN)_6_^4−^ in 0.25 m Na_2_SO_4_ solution, a 516 nm focused laser excitation (10 µW), and at 200 nm scan‐line spacings in a point‐to‐point measurement. (j) Time traces of tip current obtained from the Au NE upon local illumination of the TiO_2_ substrate and Au micro‐flake with an excitation wavelength of 516 ± 10 nm at different light intensities (2.8–82 µW). The tip was biased at 0 V vs Ag/AgCl reference electrode to operate in substrate generation‐tip collection mode. (k) Higher resolution (100 nm scan‐line spacing) point‐to‐point photo‐SECM image and the corresponding line scans of the flake edge marked with a black dashed rectangle. (l) Continuous‐scan photo‐SECM image and the full‐frame‐averaged line scans of the flake edge symbolically indicated by a red dashed rectangle in Figure 4i; the corresponding SEM and optical images are shown in Figure S34a,d. No post‐processing is applied to the spatial data.

Figure 4g shows the CV response of the studied electrodes in 50 mm H_2_SO_4_ solution. The electrodes show well‐defined gold redox peaks with a lower current value for smaller electrodes. Interestingly, a significant positive potential shift of the gold reduction peak is observed by decreasing the electrode radius from ∼550 to 75 nm, followed by a negative shift for the 45 nm‐radius NE. While the observed negative shift for the ∼45 nm‐radius NE is in agreement with the previous reports on Au NEs [76] and Ag NPs [77] and is attributed to a change in the standard potential of Au and Au oxide, we could not clarify the observed positive shifts for larger electrodes and it will be the subject of future research. Testing the electrodes in the HER regime (Figure 4h) interestingly showed that the HER onset potential also decreases with the electrode size, resulting in an enhancement of the current and thus a non‐trivial increase of the current density, i.e. surface reactivity, with size (Figure 4h).

A two‐slope HER regime was observed for sub‐100 nm NEs, i.e. the 75 and 45 nm samples, corresponding to an enhanced H adsorption before triggering of HER [78] (Figure S28k–o). All studied electrodes showed a stable voltammetry response during cycling up to the HER potential regime (Figure S28k–o), a flat CV response at high scan rates (Figure S28f–j), and a negligible (<2 times) dependency for C_dl_ to the scan rate (Figure S29b), indicating a high quality for the gold/glass interface [74]. Notably, a higher C_dl_ value was calculated for smaller size electrodes (Figure S29b), [74] in line with our observed stronger H adsorption/desorption processes that contribute in the double layer structure formation, and theoretically calculated electrode curvature/edge effects [79]. While further investigation, including theoretical modelling, will be required to clarify these observations, we propose that, in addition to size‐related effects, all the observed trends in redox potential, HER activity, and C_dl_ values may share a common origin: the varying crystallographic orientations of the electrodes [80, 81]. Smaller electrodes, with orientations closer to [111] (Figures S19 and S20b,d), are expected to exhibit a higher density of atomic interactions, which could account for the observed behavior [80]. The stability analysis on larger‐sized electrodes revealed improved stability with less recession depth (Figure S31g–i).

### 2.7 Photoelectrochemical Imaging

To further validate the functionality of our single‐crystalline Au NEs in real SECM experiments, we performed imaging of a photo‐oxidation reaction on ∼20 nm‐thick single‐crystalline Au micro‐flakes (Au MFs) on a TiO_2_ substrate [54] using a 215 nm‐radius Au NE tip (Figure S32). Specifically, we immerse the flakes in a solution of 4 mm Fe(CN)_6_^4−^ in 0.25 m Na_2_SO_4_. We electrochemically position the tip approximately 315 nm above the TiO_2_ substrate, where strong feedback happens between the tip and substrate (Figure S32c). We photoexcite the unbiased sample using a focused 516 nm laser (10 µW, backside illumination), collinear with the Au NE tip position. Under illumination, the photo‐generated hot electrons in Au MF inject into the TiO_2_ conduction band while photo‐generated holes participate in the photo‐generation of Fe(CN)₆^3^^−^ oxidant molecules [82]. By applying an oxidative potential of 0.4 V vs Ag/AgCl to the Au NE, we can exploit a competition mode of operation during irradiation, with the same oxidation reaction happening at the tip electrode and substrate surface (Figure S32a). This results in a decrease in Faradaic tip current (I_T_) on areas with higher photoactivity. As we scan the sample in a point‐to‐point manner, we can thus build an image of the sample activity with a pixel size of 200 × 200 nm^2^ (Figure 4i). We observed a fairly uniform photoactivity across the flake surface, as previously reported in dark conditions [83]. To the best of the authors’ knowledge, this is the first report demonstrating that chemically‐grown metal nanoelectrodes can be used for electrochemical imaging applications [47, 48], enabled by the reliable electrical contact of the electrodes, which results in stable, low‐noise signal detection.

Au MFs can inherently feature truncated corners and sharp V‐shaped edges, with their size depending on the growth conditions (Figure S34a,d) [54] Our high‐resolution point‐to‐point photo‐SECM imaging, performed with a 100 × 100 nm^2^ pixel size (Figure 4l), resolves fine structural details. including the sub‐200 nm truncated geometry at the corner of the studied flake. The observed lateral resolution of ∼250 nm, estimated using the 10–90% edge‐width criterion (dashed lines in Figure S35a), reflects diffusion‐induced broadening inherent to SECM. Nevertheless, it surpasses that achieved in many previously reported photo‐SECM studies [84] and is comparable to the highest spatial resolutions reported for photo‐SECM to date [85].

High‐resolution continuous‐scan photo‐SECM imaging (Figure 4l; Figure S34e), carried out in substrate‐generation/tip‐collection (SG/TC) mode at an effective pixel size of ∼5 × 5 nm^2^ on the edge of a larger Au MF, further resolves truncated corners and fine ∼480 nm features (corresponding to the primary lateral resolution; ∆x_1_ in Figure S35b) associated with sub‐200 nm sharp edges of the flake (Figure S34a). These features are detected using a 200 nm‐radius Au NE (smaller than the measured resolution), achieving a sub‐1 pA current‐step sensitivity, as evidenced by the corresponding line scan shown in Figure 4l.

In photo‐SECM experiments employing localized illumination, the effective spatial resolution can be influenced not only by the tip radius and tip‐substrate distance, but also by illumination geometry [3], carrier diffusion within the photocatalyst [3, 86, 87], photoactivity of the substrate, and photo‐induced thermal effects [88], all of which can broaden the apparent reaction zone. These effects are clearly reflected in our photo‐SECM images and the corresponding line scans, acquired using two different beam sizes (∼1.3 and 6.5 µm FHWM) and illumination powers (1 and 10 µW) while maintaining the same tip size and tip‐substrate distance (Figure S34). Increasing the beam size and laser power leads to a noticeable reduction in the secondary spatial resolution (defined by the broader current variation and second minimum in the line scan; ∆x_3_ in Figure S35b) as well as reduced image contrast. This behavior is consistent with the much shorter diffusion length of hot carriers in metals (e.g. 10 nm for 2eV hot holes) [87] relative to the optical beam size, together with an enhanced contribution from photothermal effects, highlighting the dominant role of the laser beam parameters in determining photo‐SECM spatial resolution. Additionally, in a constant‐height imaging mode we experienced no crash of the tip (i.e. the least damage to the particle) and an extremely stable performance for over 7 h of electrochemical imaging at both oxidative (Figure 4i,k) and reductive (Figures S33b and S34) potentials. The same Au NE could be reused for multiple FIB cutting/imaging cycles; in our tests, at least five reuse cycles were demonstrated (Figure S32d–f) without any observable performance degradation or change in the RG value, despite a slight increase in tip size. These excellent capabilities all originate from the thin glass wall of the electrode, which limits broadening effects on the illumination spot, [89] as well as the single‐crystalline nature of the electrode, which limits reactivity inhomogeneities across the tip, and the perfect seal at the glass/gold interface. It is to note that higher spatial resolutions under back‐illumination conditions can be readily achieved by reducing the effective beam size, employing smaller Au NEs, using semiconducting photocatalysts with longer carrier diffusion length [86] or substrates with lower photoactivity at the excitation wavelength, and adopting smaller scan step sizes.

Local photo‐SECM measurements at varying laser powers on the TiO_2_ and Au MF regions, using a 0 V vs Ag/AgCl tip potential (Figure 4j), shows the ideal operation for the Au NEs in SG/TC mode, characterized by increasingly negative photo‐induced currents with increasing laser power. Approach curve experiments in both competition and SG/TC modes further confirmed the photo‐oxidation reaction on Au MFs (Figure S33c,d). These measurements also highlight the detection sensitivity of the tip electrode. At the minimum laser power of 2.8 µW (Figure 4j), a distinguishable current difference (∆I ± σ_noise_) of 1.08 ± 0.24 pA (RMS) was observed compared to the current trace under dark conditions, which corresponds to a signal‐to‐noise ratio (SNR) of ∼4.5 at a reciprocal response time (∆t^−1^) of 1 s^−^^1^ (0.5 Hz cut‐off frequency) even in the absence of a Faraday cage [47]. This current resolution enables detection of photo‐induced chemical reactions at solely 16.5 µm concentration of Fe(CN)_6_^3−^ oxidant molecules, with a noise‐limited detection threshold around 3.67 µm, corresponding to a high sensitivity of 65.4 nA/m, and a limit of detection (LOD) of 11.0 µm for photo‐SECM measurements. This already surpasses the performance of bulk electrochemical techniques that rely on highly‐sensitive optical probes (sub‐mm) [90] and micro‐scale SECM‐AFM Pt electrochemical probes (tens of µm) [91], overall pushing the limits toward the best values reported for electrochemical methods in confined gap space (fm to pm) [5, 92, 93, 94]. Furthermore, a comparison with ∼1 µm‐radius Pt microelectrodes having almost the same LOD values (10 µm), [6] indicates a superior sensitivity for our Au NEs in confined regions, making them well‐suited for nanoscale electrochemical imaging.

To further explore the detection limit of single‐crystalline Au electrodes, we also performed CV measurements in bulk electrolytes with FcMeOH concentrations ranging from 1 µm to 1 mm. We observed sigmoidal responses even at sub‐10 µm levels (Figure S36a–c,g–i), yielding record‐low LOD values of 79.4 nm for an ∼1.3 µm‐radius Au UME and 94.0 nm for an ∼340 nm‐radius Au NE tip (corresponding to 404.8 and 101.8 nA/m sensitivities, respectively). At such low concentrations, Faradaic current detection is governed by the stochastic collision probability of redox molecules with the electrode surface, which accounts for the lower LOD observed with the larger electrodes. Interestingly, bulk‐state LOD analysis on a polycrystalline ∼1.5 µm Au UME (See Figure S36d–f), which contains a higher density of twin and grain boundaries, revealed an LOD of 187.1 nm—approximately 2.4 times higher than its single‐crystalline counterpart (Figure S36a–c). This performance degradation likely stems from elevated background capacitive charging currents [95] (2.34 pA vs. 1.57 pA for the single‐crystalline electrode) and the closer to <111> crystallographic orientation, despite the polycrystalline electrode being slightly larger in size. Importantly, when normalized to the surface area to correct for the different stochastic collision frequency of different size electrodes, our Au single‐crystalline UMEs exhibit an LOD_area‐normalized_ (14.9 nm/µm^2^) over 235 times lower than their polycrystalline Pt counterparts (ca. 3.5 µm/µm^2^), [6] indicating an exceptional sensitivity for our single‐crystalline electrodes in chemical probing. To our knowledge, such low LODs for bulk measurements have not been previously reported, and is even further close to the confined electrochemistry and spectroscopy methods. HER activity measurements further confirmed the superior surface reactivity (i.e. higher current density) of the single‐crystalline electrodes [80] (Figure S37). Overall, these first results highlight the critical role of the polyol‐grown Au NEs’ single‐crystalline surface quality in enabling highly sensitive chemical detection—independent of instrumental limitations—and point to clear opportunities for further investigation in this direction.

### 2.8 Multi‐Material and Multi‐Functional Probes

Finally, and most remarkably, we demonstrate the extraordinary versatility of the polyol‐based chemical growth approach for fabricating not only Au NEs, but also Au UMEs, platinum NEs and multifunctional NEs tailored for advanced electrochemical microscopy techniques (Figure 4c–f). For example, by simply substituting the gold precursor with chloroplatinic acid, we successfully fabricated Pt NEs under identical growth conditions (Figure 4c), showcasing the adaptability of this method. Beyond gold and platinum, this polyol‐based growth strategy holds immense potential for other metals and alloys, including Ag [96], Cu [97], Pd [98], and even Bi [99]—materials critical for electrochemical research yet unattainable via traditional laser‐pulling methods [100].

Furthermore, we demonstrate that performing a partial cross‐sectional FIB cut on disc‐shape Au NEs can expose a recessed disc‐shape nanoelectrode and result in an L‐shape tip geometry with a protruding insulating glass tip capable of simultaneously detecting atomic forces and Faradaic processes (Figure 4d). This represents a straightforward method for fabricating dual‐functional tips for combined SECM‐atomic force microscopy (SECM‐AFM), [101, 102] paving the way for advanced research that require high‐resolution constant‐distance electrochemical and tomographic imaging.

We additionally demonstrate complete chemical growth within theta glass nanopipettes having non‐symmetric geometries, which is remarkable for fabrication of high performance thin‐wall double barrel (Figure 4e) and dual (Figure 4f) metal NEs. These versatile electrodes can enable simultaneous detection of Faradaic currents alongside ionic currents, supporting concurrent analysis of surface reactivity [103], product selectivity [104], surface topography [105], pH [106], temperature [107], surface charge [108, 109], and chemical delivery [110, 111] in combined SECM, scanning ion conductance microscopy (SICM), and scanning electrochemical cell microscopy (SECCM) techniques. Until now, such combined methods have largely been limited to carbon NEs [105, 110, 112] with limited electrochemical performance for inner‐sphere reactions or relied on surface‐modified carbon NEs [113, 114] due the difficulties with fabrication of multifunctional metal NEs with the laser pulling approach [115].

Overall, the remarkable material and geometric versatility of our approach not only expands the scope of NE fabrication but also paves the way for the development of next‐generation electrochemical microscopy and innovative applications across diverse fields by bridging between electrochemistry, biology, and spectroscopy.

## 3 Conclusion

The reported template‐assisted, non‐self‐limited polyol‐based growth of single‐crystalline gold represents a significant step‐forward in the scalable fabrication of high‐quality, thin‐wall Au NEs, addressing longstanding challenges in reproducibility, performance reliability, and versatility. By combining the polyol‐based chemical growth with optimized laser pulling of borosilicate nanopipettes, we obtained excellent reproducibility (∼80%) in the fabrication of thin‐wall, single‐crystalline Au NEs with controllable sub‐100 nm radii core sizes and low RG values (∼1.6 to ∼5). The growth method also shows an extraordinary versatility for high‐throughput fabrication of other metal/alloyed NEs (e.g. Pt, Ag, Cu, and even Bi), thin‐wall UMEs, and multifunctional NEs including dual, double‐barrel, and recessed L‐shape NEs for various electrochemical probe microscopy techniques, from SECM to EC‐STM, AFM, SICM, and SECCM hybrid methods. Comprehensive structural and electrochemical analyses on disc‐shape Au NEs confirmed a gap‐free gold‐glass interface together with a unique single‐crystalline nature of the Au core, resulting in a high electrochemical quality and stability in electrolyte environments.

An interesting size dependency was observed for the electrodes: a more positive standard potential, higher C_dl_ value, and higher HER activity were reported for smaller size electrodes, the latter being of high significance for improving the signal‐to‐noise ratio in small‐size NEs. We also demonstrated the practical potential of these NEs in photo‐SECM imaging of weak photo‐oxidation reactions on single‐crystalline Au micro‐flake surfaces at outstanding detection sensitivities (65.4 nA/m) and limit of detections (11.0 µm), with ∼250 nm spatial resolution, and extremely stable performance for over 7 h of photochemical imaging. The spatial resolution was found to be illumination‐dependent under back‐illumination conditions. Our bulk electrolyte testing further showed record‐low LOD values of 79 nm, enabled by the thin‐wall architecture and single‐crystalline nature of the electrodes. Remarkably, this performance approaches the range attainable with confined electrochemistry [5, 92] or advanced spectroscopic techniques [116] (fm–pm range).

By addressing long‐standing issues in the versatile fabrication of high‐quality thin‐wall metal NEs/UMEs, this work paves the way for the development of next‐generation spectroelectrochemical and near‐field microscopies toward high‐resolution single‐molecule and single‐nanoparticle analysis. This also opens completely new possibilities in nanoelectrochemisty, material science and biochemistry by providing an excellent single‐crystalline platform for operando fundamental research into interfacial phenomena, such as double‐layer structure, [53] nanoscale charge transport mechanisms, [64] as well as single‐enzyme kinetics, [117, 118] neurotransmissions, [41] and biomolecular interactions [22].

## 4 Methods

### 4.1 Fabrication of Nanopipettes

Single‐barrel nanopipettes were fabricated from borosilicate glass capillaries (1.2 mm outer diameter, 0.69 mm inner diameter; Science Products GmbH) having no filament inside. The capillaries were cleaned by sonication in acetone and ethanol for 10 min, followed by several times rinsing with ultrapure water and drying in an oven at 100˚C before pulling. No hazardous Pirhana or silanization treatments were used. The nanopipettes were fabricated by using a combined laser shrinking and laser pulling method on a laser‐based P‐2000 pipette puller (Sutter Instruments). First, the glass capillaries were locally shrank at the center using high energy of the CO_2_ laser for increasing the wall thickness and thus the strength of the capillary before the pulling process. In this step, a pair of home‐made stoppers were used to fix the pulling arms and then to controllably soften and shrink the glass capillary with a program listed in Table S1. Next, after removing the stoppers, an optimized two‐line program (Table S1) was used to hard pull the pre‐shrank capillaries into ultrafine nanopipettes. At constant Heat parameters, the duration of the shrinking Time and the Delay parameter in the hard pulling step were separately used to control the achievable orifice size and the shank/taper length of the nanopipettes, respectively. Sub‐20 nm‐radius short‐taper nanopipettes were obtained at a > 90% repeatability by a 90 s shrinking time and a 190 Delay parameter in the hard pull step (Figure S3b). Theta nanopipettes were fabricated from borosilicate theta capillaries using the program reported in Table S2. The orifice size of the fabricated nanopipettes was characterized under SEM without using any conductive coating. Image integration at low electron beam energy, i.e. 2 kV, was used for SEM imaging with the least charging effect without using any conductive coating material.

### 4.2 Fabrication of Gold Nanoelectrodes

Gold nanoelectrodes were fabricated by a polyol‐based chemical growth process [54] inside ideal‐shape glass nanopipette templates (20 nm‐radius and 250 µm‐length taper). First, the tip of the glass nanopipette was dipped in pure EG, and then the glass nanopipette was backfilled with a growth solution containing 0.125 m HAuCl_4_ in EG. This resulted in an advantageous separation, i.e. an air bubble, between the backfilled growth solution and the tiny front‐filled EG. The filled nanopipettes were then vertically hold in 6 mL glass vials containing a bulk solution of 2 mL EG and 0.5 mL 200 mm NaBH_4_ in ethanol. Next, the vials were immediately transferred to a muffle furnace (Nabertherm GmbH) heated at 110°C for starting chemical growth for 24 h in all nanopipettes in a batch. This condition enabled selective growth of continuous ∼300 µm‐length Au deposits at an >80% repeatability with the least amount of unwanted particles and chemical etching on both exterior and interior surfaces. The NEs were then naturally cooled down in the furnace and rinsed by ultrapure water and ethanol for removing the bulk solution residues from their surface. A cleaning treatment with ultrapure water and ethanol was then performed on the as‐grown NEs in order to remove the remained growth solution and any randomly formed unwanted particles, if any, at the nanopipette neck, behind the solution/air interface of the air bubble. Next, a long‐taper W micro‐contact was physically connected to the Au deposit under an optical microscope to establish a soft electrical contact. The Au NEs were finally perfected by complete FIB cutting of the protruded Au or etched glass imperfect parts into disc‐shape electrodes. A 30 kV and 2 pA Ga ion beam was used for sharp cutting of the NEs.

### 4.3 Fabrication of Long‐Taper W Micro‐Contacts

Long‐taper W micro‐contacts were prepared by using a very facile two‐step electrochemical approach consisting of thinning and cutting steps (Figure S15). A W microwire (25 µm diameter, Goodfellow, 99.9%) was first vertically immersed by 3.5 mm into a 10 mL of 2 m KOH etchant solution using a manual microstage. In the first step, a 0.5 V DC potential was applied between the W wire and a Cu wire loop cathode 1 cm below the solution surface. This results in oxidative dissolution of WO_2_^−^ anions in water, especially slightly below the meniscus at the air‐electrolyte interface, leading to a necking shape and a long uniformly thinned section that can finally drop off at the neck in a complete etching process [119]. The etching process was controlled by monitoring the current level until a ∼50% current drop was observed, and stopped by stopping the bias. At this step, the W micro‐wire is incompletely etched and thinned down to an ∼5 µm diameter. Then, the etched W wire was lifted up by ∼1 mm and biased at a lower potential, e.g. 0.2 V. An ∼700 µm‐length W wire was cut and tapered at this step after the drop off of the bottom part controlled by a sudden current drop. The fabricated W micro‐contact was finally rinsed with DI water to terminate the etching reaction. In this method, the current drop criteria at the first step and the amount of the upward lift at the second step separately allow a good control over the width and the taper length of the W micro‐contact, respectively. An > 90% reproducibility was achieved for fabrication of ideal‐profile (5 µm diameter 700 µm taper length) W micro‐contacts for Au NEs.

### 4.4 Electrochemical Testing of Gold Nanoelectrodes

All voltametric experiments were performed in a one‐compartment three‐electrode cell connected to a CHI 760E potentiostat (CH Instruments) without any Faraday cage, unless otherwise noted in the figure captions. A leak‐free Ag/AgCl reference electrode and an Au counter electrode were used to avoid cross‐contamination of the Au NEs with Ag^+^/Cl^−^ ions and/or Pt during electrochemical testing. To remove oxygen, the electrolyte solutions were purged with N_2_ gas for 30 min before the experiments. First, outer‐sphere voltammetry was performed in a 2 mm Fe(CN)_6_^4−^/0.25 m Na_2_SO_4_ and/or 1 mm FcMeOH/0.125 m KCl solutions for evaluation of the electrical connection and geometrical surface area. The effective radius of the electrodes was evaluated from the measured diffusion‐limiting currents through the modified Saito's equation [67, 68] (see Equation 1) for different RG values estimated from SEM imaging. Next, the electrodes were activated by multiple cycling in 50 mm H_2_SO_4_ solution within a 0 to 1.4 V vs Ag/AgCl potential window at 100 mV.s^−1^ scan rate for about 30 cycles until a pristine surface was produced. Cycling was stopped once a stable CV response was observed. Steady‐state voltammetry of the electrodes was measured at a 10 mV.s^−1^ scan rate within 0.2 to 1.4 V and −0.8 to 0.8 V vs Ag/AgCl potential windows for evaluation of the Au redox processes and HER activity, respectively. The electrochemical surface area and roughness factor (RF) of the electrodes were calculated from the characteristic values of the consumed charge for the reduction of an AuO monolayer in a one‐to‐one ratio (390 µC.cm^−2^) and the ratio of the calculated microscopic surface area to the geometrical area (A_m_/A_g_), respectively [62]. The double‐layer charging current, i_c_, was background subtracted for the surface area calculations due to the low current level of the Au redox process on NEs. The sealing quality of the electrodes was electrochemically characterized by performing CVs in the double layer and HER regimes and evaluation of the CV profiles [74]. Double layer capacitance (C_dl_) values in F cm^−2^ were obtained by calculating the i_c_ at 0.3 V vs Ag/AgCl from double layer CVs and by using C_dl_ = |∆i_c_|/2A_m_.v, where A_m_ is the microscopic area of the electrode in cm^2^, and v is the scan rate in V s^−1^. Unless otherwise specified, all the experiments were repeated three times, and only the second cycles were reported for ensuring a steady‐state response condition. A fast Fourier transform (FFT) filter was used for smoothing the high‐scan rate measurement data.

Singal‐to‐noise ratio (SNR) for nanoelectrodes was calculated by dividing the light‐induced change in tip current, i.e. current step amplitude (Δ*I*), to the corresponding standard deviation of the measured background current noise, i.e. noise level (σ_*noise*_), based on the measurements reported in Figure 4j. To reduce high frequency environmental noise, a 0.5 Hz low pass filtering was applied to the measurement (∆*t* = 1/2*f* = 1s). Detection sensitivity (*S*) and limit of detection (LOD) were obtained by using the modified Saito's Equation (Equation 1) for C^*^ calculation, and the following Equations (3) and (4), [91]

(3) $$S=\frac{\Delta I}{C^{*}}$$

(4) $$LOD=\frac{3\sigma_{noise}}{S}$$

where *S* is determined from the slope of the Δ*I* vs. C* curve, derived from data measured at different laser powers and concentrations.

### 4.5 Synthesis of Gold Micro‐flakes

High‐quality single‐crystalline gold micro‐flakes were directly grown on borosilicate glass substrates by a halide and gap‐assisted polyol process [54]. A PMMA wet‐transferring method [120] was used for transferring the Au MFs onto a TiO_2_/ITO‐coated glass substrate. The sample was then exposed to an oxygen plasma (2 min, 500 W; Tepla 300) to remove any PMMA residue left from the transferring step.

### 4.6 Transmission Electron Microscopy

TEM analysis was performed using a FEI Tecnai Osiris microscope operated at 200 kV. BF‐TEM images were recorded on a Gatan Orius camera. Crystal orientation mappings were performed using an Astar system [60]. Specifically, an electron probe of 14 pA was precessed on the sample at an angle of 1° at 100 Hz and raster‐scanned with a 2.5 nm step size and 50 ms dwell time. Local precession‐assisted diffraction patterns were collected with a Stingray camera recording the phosphor screen at a camera length of 165 mm. The Astar v2.2 software suite was used to index the pseudo‐kinematic diffraction patterns of the Au face‐centered cubic material. Finally, the data representation and analysis used MTEX v5.10.2 toolbox for MATLAB [121].

### 4.7 Scanning Photo‐Electrochemical Microscopy

Photo‐SECM imaging was performed using a home‐made SECM instrument on an inverted optical microscope for back illumination of the sample (Figure S32a). Measurements were done in a three‐electrode cell containing a leak‐free Ag/AgCl reference electrode and a Pt counter electrode in a 4 mm Fe(CN)_6_^4−^ in 0.25 m Na_2_SO_4_ electrolyte solution. Tip Z scanning and sample XY scanning were realized by using Nano‐OP and Nano‐LP XY piezo‐stages (Mad City Labs). Electrochemical measurements were done by a VSP300 potentiostat (Biologic). The Au NE tip was biased at a diffusion‐limiting 0.4 V vs Ag/AgCl potential, and positioned at a distance corresponding to a 25% setpoint value in a negative feedback approach curve. An approach curve fitting was used by employing analytical approximations reported in [122]. Constant‐height point‐to‐point SECM imaging was performed by illumination of the sample with a 516 nm focused laser (10 µW) and applying an oxidative tip potential, i.e. 0.4 V vs Ag/AgCl, which satisfies a competition mode of operation for a photo‐oxidation reaction on the substrate. The beam size was about 1.3 µm, and the sample was scanned at 200 nm step sizes using an acquisition time of 500 ms per pixel. All the data were recorded using in‐house programs written in LabView (National Instruments), and were plotted with Origin Pro software with no extra smoothing method. Continuous‐scan photo‐SECM images were obtained by an FPGA controller with a 1 µs sampling rate and using a 200 nm‐radius Au NE at a 16 and 1 µm.s^−1^ scan speeds and image sizes of 256 and 2048 pixels for the full‐scan and zoomed images, respectively.

## Conflicts of Interest

The authors declare no conflict of interest.

## Supporting information




**Supporting File**: smll72948‐sup‐0001‐SuppMat.pdf.

## Data Availability

The data that support the findings of this study are available in the supplementary material of this article.
