# Supplementary material for: Thin‐Wall Single‐Crystal Gold Nanoelectrodes toward Advanced Chemical Probing and Imaging
Source: Small. 2026 Feb 26;22(23):e14938. doi: 10.1002/smll.202514938 (PMC13100563; doi:10.1002/smll.202514938)
Supplement: Supplementary file 1 — Supporting File: smll72948‐sup‐0001‐SuppMat.pdf. [file SMLL-22-e14938-s001.pdf]

Thin-wall Single-crystal Gold Nanoelectrodes towards Advanced Chemical Probing and Imaging

Authors: Milad Sabzehparvar[1], Fatemeh Kiani[1], Germán García Martínez[1], Omer Can Karaman[1], Victor Boureau[2], Lucie Navratilova[2], Giulia Tagliabue[1*]

[1] Laboratory of Nanoscience for Energy Technologies (LNET), STI, École Polytechnique Fédérale de Lausanne, 1015 Lausanne, Switzerland

[2] Interdisciplinary Center for Electron Microscopy (CIME), École Polytechnique Fédérale de Lausanne, 1015 Lausanne, Switzerland

*E-mail: giulia.tagliabue@epfl.ch

Contents

Supplementary Information 1 – Thin-wall Disc-shape Nanoelectrodes/Microelectrodes

Supplementary Information 2 – Fabrication of Nanopipettes

Supplementary Information 3 – Fabrication of Au Nanoelectrodes

Supplementary Information 4 – Optimization of Growth Condition

Supplementary Information 5 – Bipolar Electrochemical Contacting

Supplementary Information 6 – Electrochemical Sharpening of Long-taper W Micro-contacts

Supplementary Information 7 – Material Characterization

Supplementary Information 8 – Electrochemical Characterization of Au Electrodes

Supplementary Information 1 – Thin-wall Disc-shape Nanoelectrodes/Microelectrodes

Thin-wall nanoelectrodes/microelectrodes (NEs/UMEs) are generally considered as disc-shape electrochemical probes surrounded with an insulating sheath having an insulator-to-conductive core radius ratio (known as the RG value) smaller than 10, where back-diffusion of chemical species from behind the insulating sheath gets more significant. Despite the widespread use of the term "thin-wall" or "low-RG-value" electrodes, there is no consensus on what constitutes a critical RG value to define a thin-wall electrode. To address this, we numerically calculate the mass transport of chemical species for electrodes with varying RG values. To model the tip response (i_T, $C_{glass/electrolyte}$, and J_z) for a 100 nm-radius nanoelectrode in bulk electrolyte media, a 2D axisymmetric simulation was performed using COMSOL Multiphysics v5.6.[1,2] The steady-state diffusion of the redox species (i.e. 2 mM $Fe(CN)_6^{3-/4-}$) in this model was simulated using the transport of diluted species (tds) module and solving the differential equation relating to the Fick's second law of diffusion in cylindrical coordinate,

$$\frac{\partial^2 c_i}{\partial r^2} + \frac{1}{r}\frac{\partial c_i}{\partial r} + \frac{\partial^2 c_i}{\partial z^2} = 0; 0 \le r \le r_s, 0 \le z \le l \quad \text{(S1)}$$

where r and z are the coordinates directions, and r_s and l are the width and the height of the simulation space, respectively. c_i in this model is the concentration of reductant (*Red*) for a diffusion-controlled oxidation reaction at the tip electrode.

$$Fe(CN)_6^{4-}\ (Red) - e^- \rightarrow Fe(CN)_6^{3-}\ (Ox) \quad \text{(S2)}$$

Figure S1a,b shows the simulated tip current value (i_T) at the conductive core and local concentration of Red at the glass/electrolyte interface for 100 nm-radius nanoelectrodes having different RG values, from 1.2 to 20. As evident, both the tip current and concentration values start to deviate for electrodes having RG values smaller than 10 due to stronger back-diffusion of *Ox* species towards the conductive core. Interestingly, intercept of slopes for the i_T versus RG curve reveals an optimum RG value of 1.8 (~2) which corresponds to approximately the same glass wall thickness to the conductive core radius for the electrodes. The concentration profile and streamlines of *Red* species for electrodes having 1.2, 2, and 5 RG values in **Figure S1d-f** visually show the back-diffusion conditions. Positive flux distribution of *Red* species over the tip surface (see **Figure S1c**) indicates a general increasing trend for back diffusion by decreasing the RG value. Interestingly, both the negative flux at the core/insulator interface and the flux at the core surface increase by decreasing the RG value.

In summary, thin-wall NEs with an optimum RG value of 2 or smaller provide higher levels of Faradaic current, higher normal diffusive fluxes, and smaller overall dimensions, enabling higher signal-to-noise ratios, chemical detection sensitivity, and spatial resolution for electrochemical research, respectively.

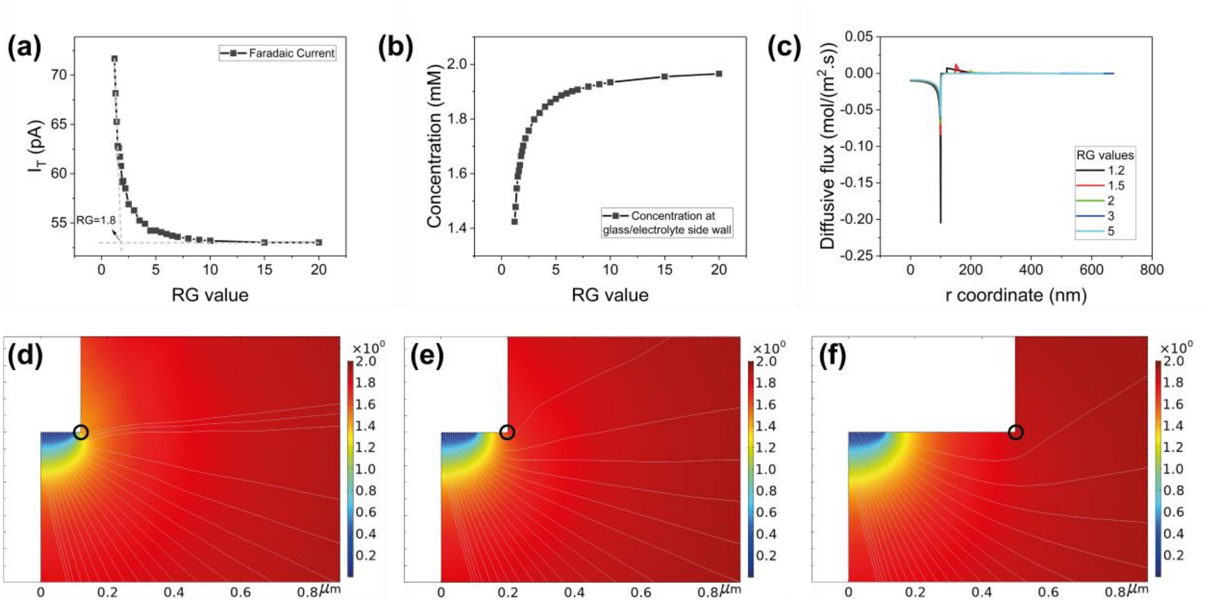

Figure S1. The effect of RG value on electrochemical response of 100 nm-core radius nanoelectrodes. Plots of theoretically calculated Faradaic tip current versus RG value (a) and back-side concentration versus RG value (b) for a 100 nm-radius tip electrode having different glass sheath sizes. The concentration in (b) is the value calculated at the black circles in panels (d-f) at the glass/electrolyte interface. (c) Distribution of the normal diffusive flux over the tip surface and electrolyte media for nanoelectrodes having different glass sheath sizes. Theoretically calculated axisymmetric concentration profiles of reductant molecules for nanoelectrodes having different RG values of 1.2 (d), 2 (e), and 5 (f) biased at a mass-transport limiting oxidative potential.

Supplementary Information 2 – Fabrication of Nanopipettes

Ideal-shape nanopipettes with sub-20 nm radius orifice sizes and ~3 mm shank length (200-300 µm taper length) were fabricated from without-filament thin-wall glass capillaries (1.2 mm outer diameter, 0.69 mm inner diameter; Science Products GmbH) using an advanced pulling approach (see **Figure S2**). Without-filament capillaries were used to prevent evaporation of the growth solution and decrease the formation of nanoparticles on the exterior surfaces during the Au NE fabrication process (see **Supplementary Information S3**). A key step is the *laser-assisted pre-shrinking process*, in which a cleaned glass capillary is locally thickened at the center by controlled irradiation ("Laser Shrinking" program, **Table S1**). This treatment overcomes the intrinsic softness problem of borosilicate glass by locally increasing wall thickness, enabling the fabrication of ultrasmall nanopipettes with high mechanical stability. The extent of shrinking is determined by the applied heat input and irradiation time. After shrinking, the stoppers are removed and the capillary is pulled into two identical nanopipettes using a delayed two-line "Hard Pulling" program (**Table S1**). At optimized Heat values, the Delay parameter (the time between laser irradiation and pulling) directly controls the shank length. This combined shrinking–pulling process allows fine control over orifice size, RG value, and taper length with ~90% reproducibility. For example, for a fixed taper length of ~250 µm, longer shrinking times yield smaller orifice sizes, provided that Heat and Delay parameters are adjusted accordingly (**Figure S2g**). Generally, higher Heat input and shorter Delay values are required for thicker walls generated by longer shrinking times. Increasing the Heat value in the shrinking step can result in much smaller nanopipettes and larger RG values in shorter shrinking Times (see **Figure S3**). Importantly, this strategy enables fabrication of short-taper nanopipettes with large RG values (e.g., ~6; **Figure S3d**) that are not available commercially, while maintaining precise control over nanoscale orifice dimensions (**Figure S2c–f**).

Compared to previously reported secondary post-processing methods such as electron-beam shrinking[3] or atomic layer deposition of insulating coatings[4], this direct approach provides more reliable and reproducible control of nanopipette geometry. **Figure S2b** shows a typical "ideal" nanopipette geometry used in this work.

Table S1. Parameter for fabrication of 20 nm-radius short-taper borosilicate glass nanopipettes.

Step		Heat	Filament	Velocity	Delay	Pull	Time
Laser Shrinking		550	5	12	120	0	90 s (variable)
Hard Pulling	Line 1	440	3	30	220	0	NA
	Line 2	450	4	40	190	255	8-9 s (laser on)

Table S2. Parameter for fabrication of borosilicate glass theta nanopipettes.

Step		Heat	Filament	Velocity	Delay	Pull	Time
Hard Pulling	Line 1	460	3	30	220	0	NA
	Line 2	490	4	45	160	250	8-9 s (laser on)

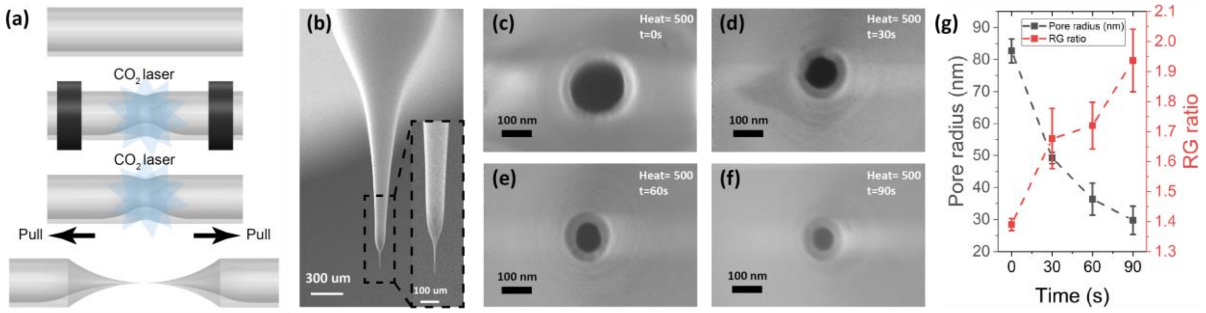

Figure S2. Glass nanopipette fabrication. (a) Schematic of the advanced pulling approach consisting of laser shrinking and hard pulling steps. (b) SEM image of a typical peak-shape glass nanopipette with a short taper. (c-f) Top-view SEM images of the nanopipettes fabricated at different laser shrinking Times of 0 s to 90 s at the same Heat value of 500. (g) Plot of pore radius and RG ratio as a function of shrinking time at Heat value of 500 and for an ~250 μm taper length. The values were calculated from SEM images of three nanopipette samples.

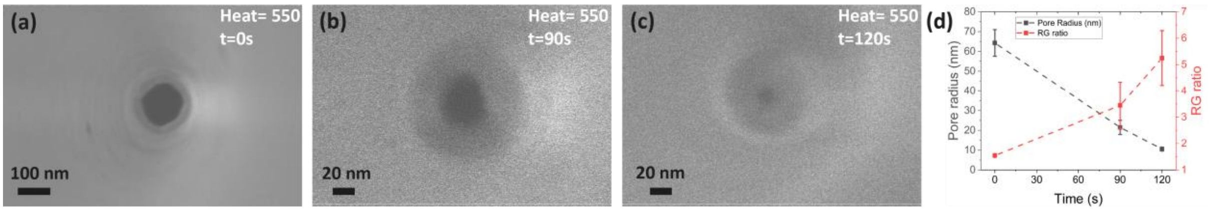

Figure S3. SEM image of the glass nanopipettes fabricated at different laser shrinking Times of 0 s (a), 90 s (b), and 120 s (c) at the same Heat value of 550. (d) Plot of pore radius and RG ratio as a function of shrinking time at Heat value of 550 and for an ~250 μm taper length. The values were calculated from SEM images of three nanopipette samples.

Supplementary Information 3 – Fabrication of Au Nanoelectrodes

Gold NEs were fabricated by a polyol-based chemical growth process[5] inside ideal-shape borosilicate glass nanopipette templates (20 nm-radius and 250 μm-length taper; See **Figure S3b**). A special filling procedure was found ideal for injecting the growth solution inside the nanopipettes. First, the glass nanopipette tips were dipped in pure EG. Next, the glass nanopipettes were backfilled with a growth

solution containing 0.125 M $HAuCl_4$ in EG using a home-built glass injector. No centrifugation force[6] or microwave heating[7] was used for complete filling of the nanopipettes due to their role in the formation/accumulation of unwanted nanoparticles across the narrow channel taper of the nanopipette that would otherwise make different nucleation sites and discontinuity in the Au deposit during the final growth process. This procedure resulted in the presence of a small air bubble between the backfilled high-concentration solution and the tiny front-filled EG solution at the bottom of the nanopipette, which beneficially avoids rapid mixing and homogenization of the $AuCl_4^-$ ions concentration and helps with the prevention of unwanted Au particles formation on both the exterior surfaces and the narrow section of the nanopipettes during the growth process.

The filled nanopipettes were then vertically hold in 6 mL glass vials (Infochroma AG) containing a bulk solution of 2 mL EG and 0.5 mL 200 mM $NaBH_4$ in ethanol. Small pieces of silicone tubes were used for holding the nanopipettes inside the vial caps. Next, the vials were rapidly transferred to a muffle furnace (Nabertherm) heated at 110 ˚C for the 24h chemical growth. In this condition, $AuCl_4^-$ ions are gradually transported from the nanopipette stem (high concentration) towards the opening (low concentration) due to diffusion and convection. At the nanopipette tip there is the highest physical confinement effect and the highest concentration of the strong reducing agent BH_4^- ions for gold nucleation. This results in single-crystalline growth of multiple twinned Au seeds at the nanopipette orifice that eventually block the channel and continue to grow upward by −CHO reducing ions from the heated EG inside the electrode[5]. This condition enables selective growth of continuous ~300 µm-length Au deposits at an > 80% repeatability with the least amount of unwanted gold particles and glass etching on the exterior surfaces (**Figures S4** and **S5a,b**). The NEs were then naturally cooled down in the furnace and rinsed with ultrapure water and ethanol for removing the bulk solution from their surface. It is to note that the as-grown NEs must not be kept in the bulk solution for long time at room temperature in order to prevent the huge precipitation of an unknown deposit on their surface. A cleaning treatment consisting of consecutive cycles of water and ethanol injection and removal was then performed on the as-grown NEs in order to remove the unwanted particles that are randomly formed inside the nanopipette body. A micro-capillary glass nozzle connected to a weak vacuum pump was used for this purpose.

Next, a long-taper W micro-wire was physically connected to the Au deposit under an optical microscope (Nikon YM-EPI) to establish a soft electrical contact (see **Supplementary Information S6**). A double-side tape and an epoxy resin (Torr Seal; Varian Vacuum Technologies) were used for temporarily fixing the nanoelectrode on a glass coverslip and permanently fixing the electrical connection. The Au NEs were finally perfected by FIB cutting (Zeiss CrossBeam XB540) the protruded Au or etched glass imperfect parts into disc-shape electrodes (**Figure S5c,d**). **Figure S7** shows multiple NEs on a FIB/SEM stub for facile FIB cutting of multiple electrodes. A 30 kV and 2 pA (or 1pA) Ga ion beam was used for sharp cutting of the NEs. It is to note that even a 1 pA/30 kV Ga ion beam could severely etch away the thin glass wall of the NEs for relatively long exposure times, further challenging the realization of sub-50 nm radius metal NEs. Using higher RG value glass nanopipettes could effectively decrease this difficulty.

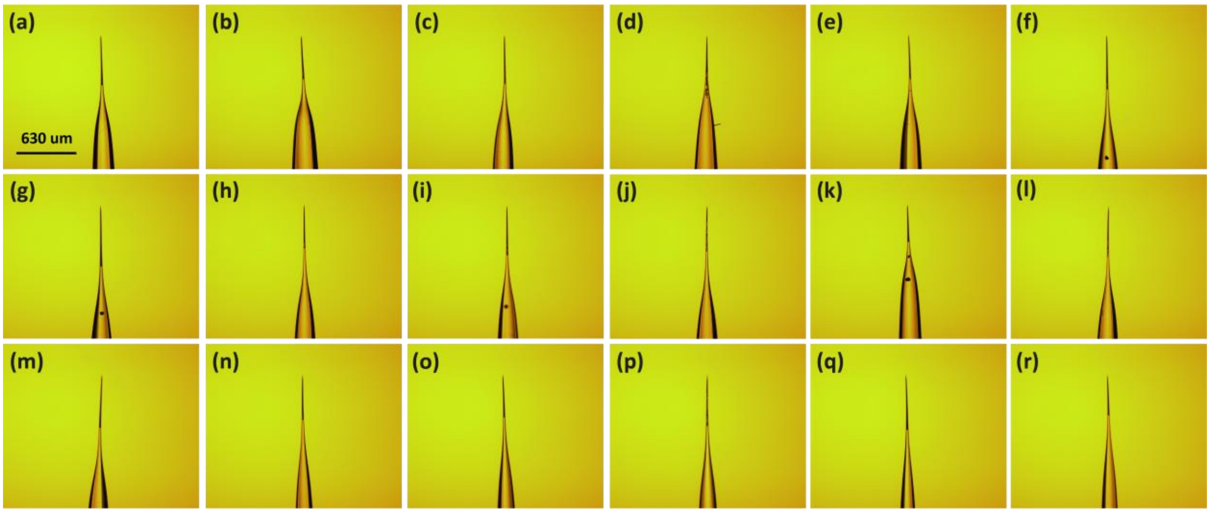

Figure S4. Reproducibility of the Au NE fabrication process at the optimized condition. The optimized condition consisted of 0.125 M $HAuCl_4$ in EG growth solution, 2 ml EG/0.5 ml 200 M $NaBH_{4,ethanol}$ bulk solution, 110 ˚C growth temperature, and 24 h growth time.

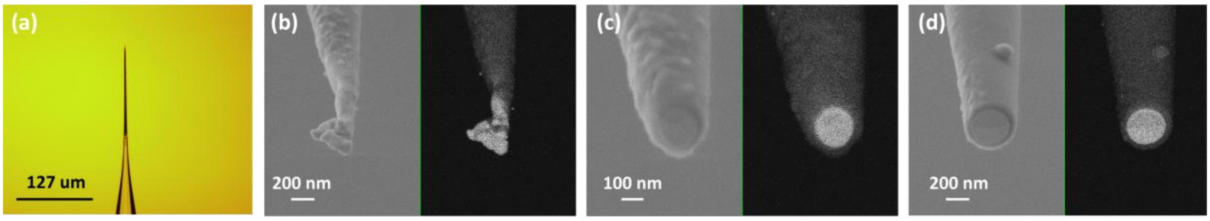

Figure S5. A typical Au NE obtained at the optimized condition. Optical micrograph (a) and SEM images of the (b) as-grown Au NE, and after a 1st and 2nd step FIB cutting process. The optimized condition consisted of 0.125 M $HAuCl_4$ in EG growth solution, 2 ml EG/0.5 ml 200 M $NaBH_{4,ethanol}$ bulk solution, 110 ˚C growth temperature, and 24h growth time.

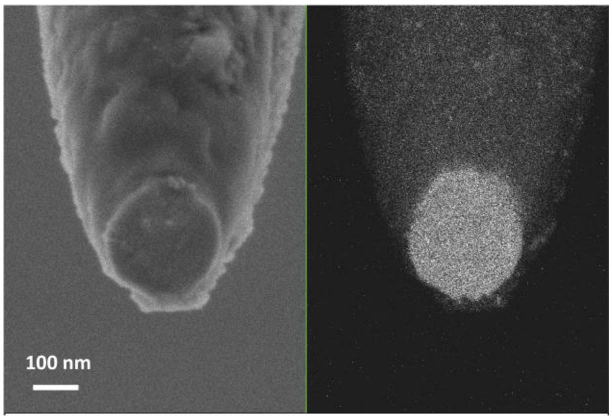

Figure S6. SEM image of an as-grown disc-shaped Au NE obtained under optimized growth conditions, requiring no additional FIB cutting.

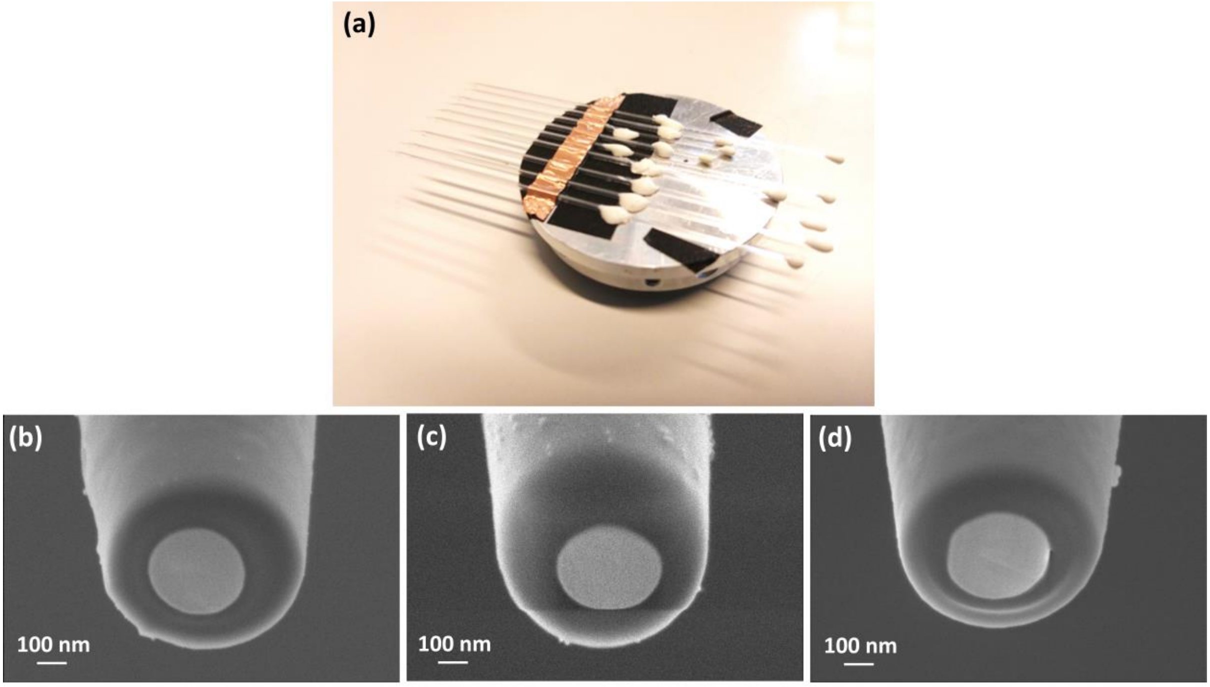

Figure S7. (a) Multiple nanoelectrodes on a FIB/SEM holder for high-throughput FIB cutting of the electrodes; (b-d) Controllable fabrication of disc-shape Au NE/UMEs by controlled FIB cutting for an specific electrode radius. SEM images of three representative Au NEs with ~175 nm core radii.

Supplementary Information 4 – Optimization of Growth Condition

To identify the optimal growth conditions, we performed several parametric studies varying the chemical media, the chemical concentrations and process time.

Our controlled experiments in different bulk media of air, pure ethylene glycol, and ethylene glycol containing different concentrations of $NaBH_4$ (see **Figure S8**) show that, instead of a random formation across the whole inner surface, gold nucleation and growth is always initiated via a space confinement effect at the very end of the nanopipette, similar to the reports for gap-assisted chemical vapor deposition growth of β-In_2S_3[8] and amorphous carbon[9,10] within mica substrates and quartz nanopores, respectively. After blocking the glass nanopipette orifice, the Au deposit continues to grow within the nanopipette. Moreover, the growth of larger size Au deposit for higher $NaBH_4$ concentrations suggests the diffusional transport of the BH_4^- reducing ions through the nanopipette orifice during the growth process before the complete blockage of the channel by gold, and their accelerating role for the crystal growth rate. We found that the concentration of BH_4^- ions not only controls the obtainable size of the Au deposit, but also the selectivity/continuity of its formation within the nanopipette channel; Lower BH_4^- concentrations resulted in formation of discontinuous and shorter Au deposits.

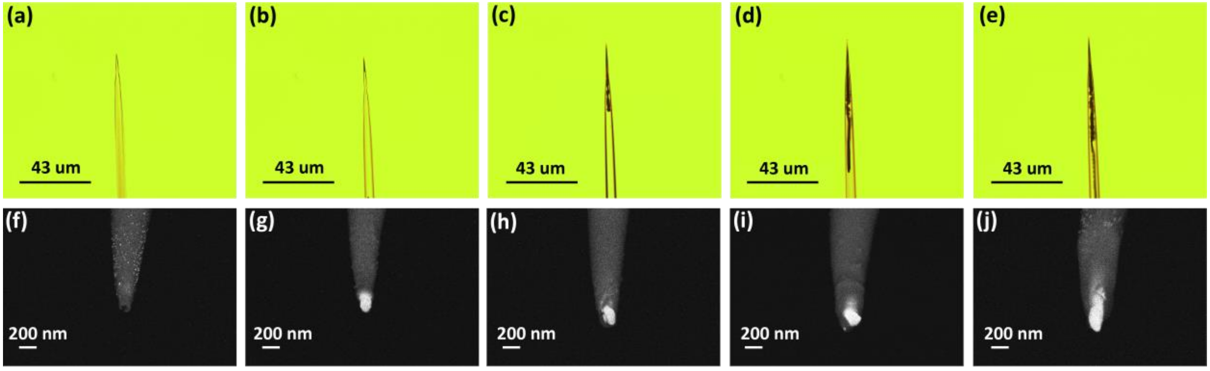

Figure S8. The effect of bulk media. Optical micrographs and SEM images of Au NEs fabricated in different bulk media of air (a,f), pure ethylene glycol (b,g), and ethylene glycol containing 100 (c,h), 300 (d,i), and 500 µl (e,j) of 200 mM $NaBH_4$ in ethanol. Experiments were performed with a growth solution of 10 µl of 0.5 M $HAuCl_{4,aq}$ in 1 ml EG at 110 ˚C for 24h.

Our systematic study on the effect of $AuCl_4^-$ ion concentration (see **Figure S9a-e**) also revealed that the crystal growth is very concentration-dependent resulting in a self-terminating growth, wire-like helical growth, and complete conical growth for low (0.0005-0.001 M), medium (0.0625-0.125 M), and high (0.25-0.5 M) concentrations, respectively. On the other hand, large amount of unwanted gold nanoparticles form on the exterior surface of the nanopipette for high $AuCl_4^-$ ion concentrations (see **Figure S9f-j**), revealing that the Au ions also diffuse out of the nano-orifice during the growth process in a reverse direction to the transport of BH_4^- ions. These unwanted NPs limit the realization of small-size disc-shape NEs. Therefore, as discussed above, to combine the benefits of low and high $AuCl_4^-$ concentrations (no unwanted particles and long gold deposit, respectively), we finally exploit a time-dependent variable concentration growth condition (**Figure S10b,d**). The nanopipette tip is dipped in pure EG, and then a high $AuCl_4^-$ concentration solution is back filled. In this condition, in the beginning of the growth process $AuCl_4^-$ are present in minimal amounts and cannot contribute to the massive growth of undesirable nanoparticles on the exterior of the nanopipette. As ions gradually transport from the nanopipette stem towards the opening due to diffusion and convection, they can be reduced by counter diffusing BH_4^- ions, and favor the growth of a very long Au deposit instead. The morphological change of the gold deposit across the nanopipette channel is an indication of the crystal growth in a variable $AuCl_4^-$ ion concentration condition (see **Figure S10b** and **Figure 2c**).

Our concentration studies also indicate that the tip of borosilicate glass nanopipette faces a severe chemical attack/etching at both higher $AuCl_4^-$ ions and $NaBH_4$ concentrations (**Figures S8** and **S9**), and this unwanted effect is strongly enhanced by increasing the growth temperature and time (**Figure S11**). This badly affects the gold/glass interface seal quality eventually requiring a FIB cut at higher sections and limiting the achievement of ultra small NE sizes.

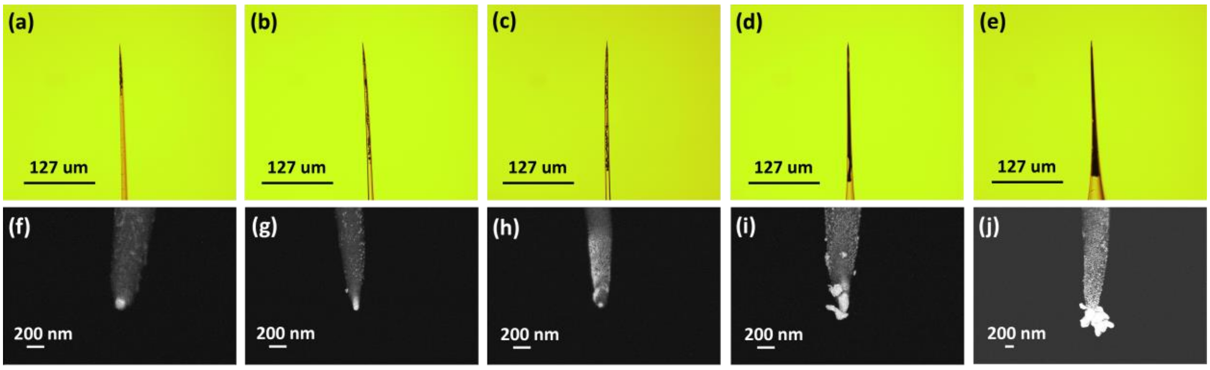

Figure S9. The effect of gold ion concentration. Optical micrographs and SEM images of Au NEs fabricated at different $AuCl_4^-$ concentrations of 0.001 M (a,f), 0.0625 M (b,g), 0.125 M (c,h), 0.25 M (d,i), and 0.5 M (e,j) $AuCl_4^-$ ions in EG. Experiments were performed within a bulk solution containing 2 ml EG and 0.5 ml 200 M $NaBH_4$ in ethanol at 110 ˚C for 24h.

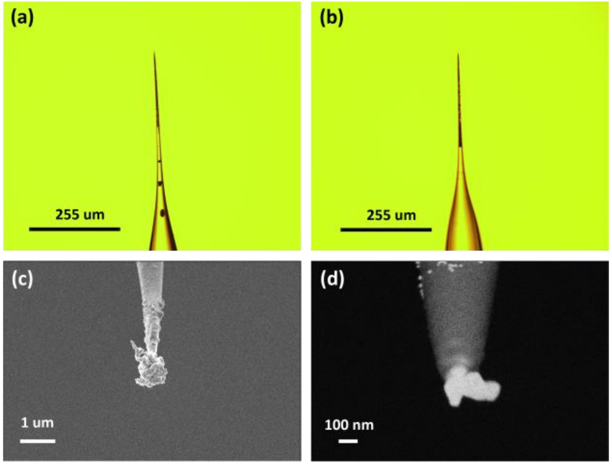

Figure S10. The effect of filling method for growth solution inside nanopipettes. (a,c) Optical micrograph and SEM image of a typical Au NE obtained by complete filling of the growth solution using a high centrifugation force at room temperature after back-filling of the nanopipette. (b,d) Optical micrograph and SEM image of a typical Au NE obtained by wetting the tip of the nanopipette by pure EG and back-filling the growth solution with a trapped air-bubble at the neck. Experiments were performed with a 0.25 M $HAuCl_4$ in EG growth solution at 110 ˚C and 2 ml EG/0.5 ml 200 M $NaBH_{4,ethanol}$ bulk solution for 24h.

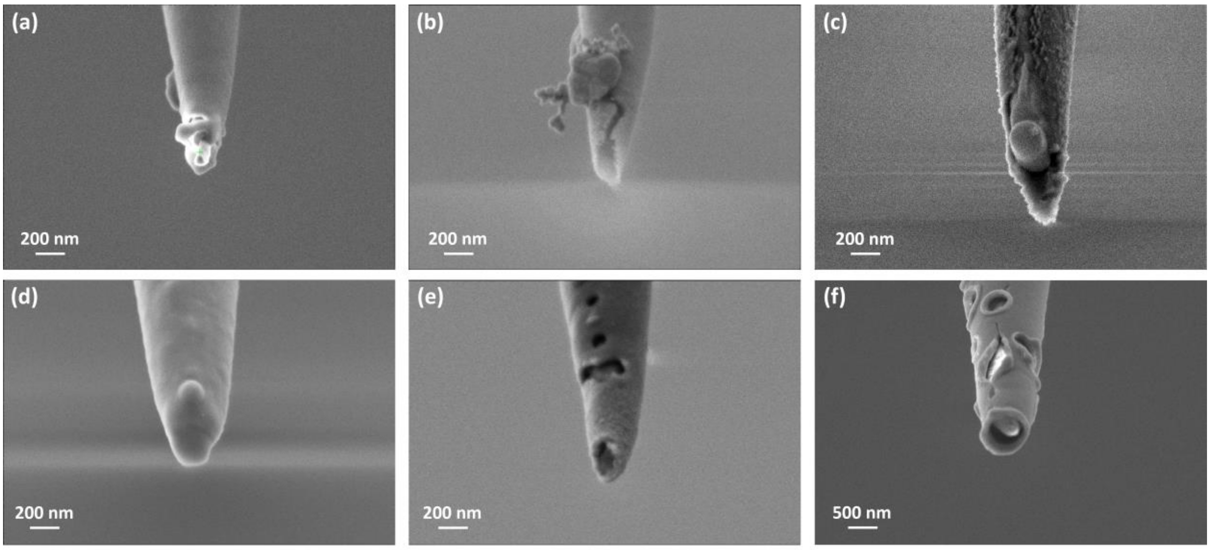

Figure S11. The effect of growth temperature and time. SEM images of Au NEs fabricated at (a) 24 h, (b) 36 h, and (c) 48 h growth times, and (d) 110 ˚C, (e) 120 ˚C, and (f) 130 ˚C growth temperatures. The experiments were performed with a growth solution of 0.5 M $HAuCl_4$ in EG inside a bulk solution containing 2 ml EG and 0.5 ml 200 M $NaBH_4$ in ethanol.

Supplementary Information 5 – Bipolar Electrochemical Contacting

The fabricated Au NEs were tested by a bipolar electrochemical contacting approach using a Ag/AgCl wire wirelessly (i.e. ionically) connected to the Au deposit through a conductive electrolyte containing 10 mM $Fe(CN)_6^{4-/3-}$ in 0.125 M KCl aqueous solution.[11,12] In the bipolar NE system, a transient redox reaction on the exterior surface of the Au deposit is detected by a reverse reaction on its interior interface and an ionic flow to the Ag/AgCl wire[13] (see **Figure S12a**). But this bipolar process can happen only if there is no gap at the Au/glass interface for ionic flow. **Figure S13** shows the electrochemical performance of the bipolar Au NEs having different nanometric gap sizes at the Au/glass interface tested in a 2 mM $Fe(CN)_6^{4-/3-}$ in 0.125 M KCl solution, showing linear, rectified and sigmoidal CV responses. CV testing on perfect bipolar NEs showed the same sigmoidal-shape CV even after 20 cycles (see **Figure S12c**), but a poor long-term stability after 4 hours of storage with the backfilled electrolyte (see **Figure S12d**), with a positive rectification effect in the -0.4 to 0 V vs Ag/AgCl potential window due to the formation of an open nanometric gap at the gold/glass interface and ionic current leakage. The observed instability is due to the reported instabilities of gold in the presence of $Fe(CN)_6^{4-/3-}$ and Cl^- ions.[14,15] Moreover, our experiments at different bulk electrolyte concentrations revealed a Faradaic current saturation, i.e. current-concentration nonlinearity, and a capacitive current enhancement for higher bulk electrolyte concentrations (see **Figure S12b**). This is an intrinsic limitation with bipolar NEs and originates from the limited mass transport and reaction kinetics at the interior Au interface.[12] These observed shortcomings of the bipolar contacting approach do not satisfy the main requirements for the electrochemical performance of NEs in most of the applications, necessitating a standard physical contacting method for practical realization of chemically grown Au NEs. However, this has remained challenging so far due the self-terminating growth process by the previous reports[11,16,17] and the lack of a facile method for preparation of soft metallic micro-contacts having long tapers for safe electrical connection within the very fragile narrow channels of the NEs[18].

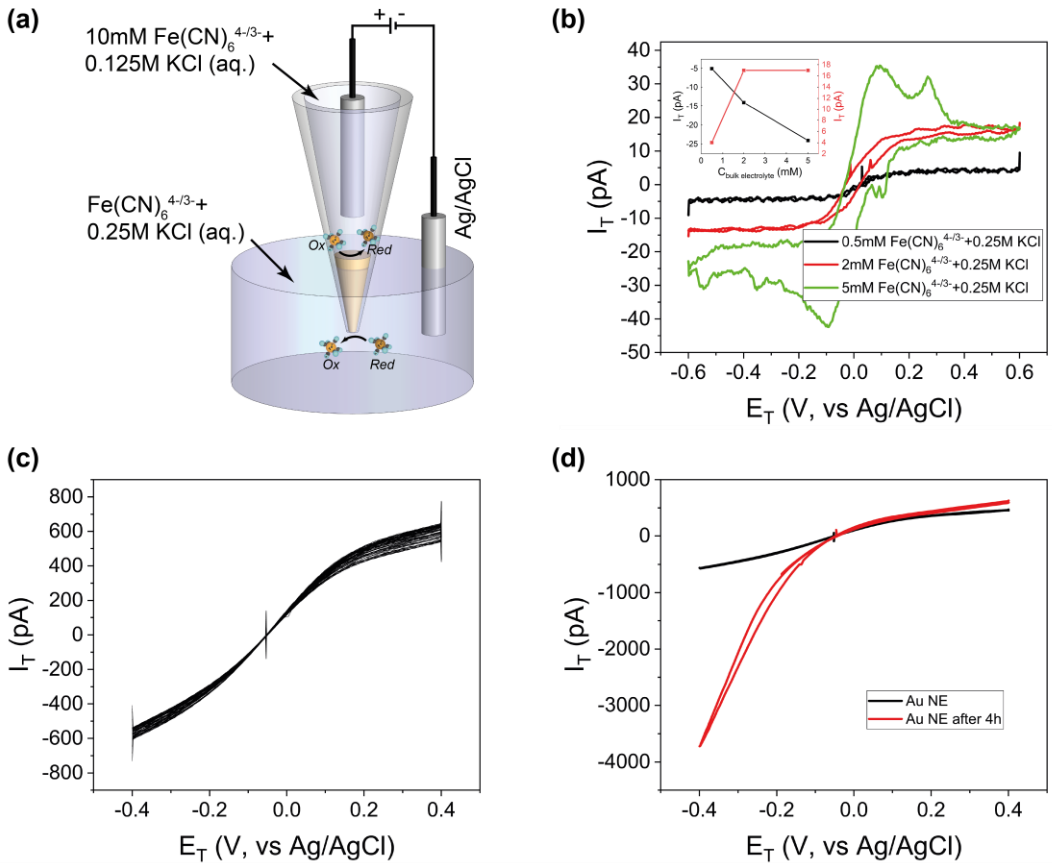

Figure S12. Bipolar electrochemical contacting of Au NEs. (a) Schematic of the bipolar NE tested in a two-electrode configuration with an Ag/AgCl wire inside an electrolyte solution containing 10 mM $Fe(CN)_6^{4-/3-}$ in 0.125 M $KCl_{aq.}$ within the NE channel and another Ag/AgCl wire in the bulk solution containing $Fe(CN)_6^{4-/3-}$ in 0.125 M $KCl_{aq.}$. (b) Cyclic voltammogram of a 20 nm bipolar NE in different bulk electrolyte concentrations of 0.5 mM, 2 mM, and 5 mM $Fe(CN)_6^{4-/3-}$ in 0.125 M $KCl_{aq.}$. Inset shows the steady-state current-concentration profile for both the oxidation (red) and reduction (black) reactions. (c) Multi-cycle voltammetry for short-term stability test on a bipolar Au NE. (d) Voltammetry for long-term stability test on a bipolar Au NE after 4 hours.

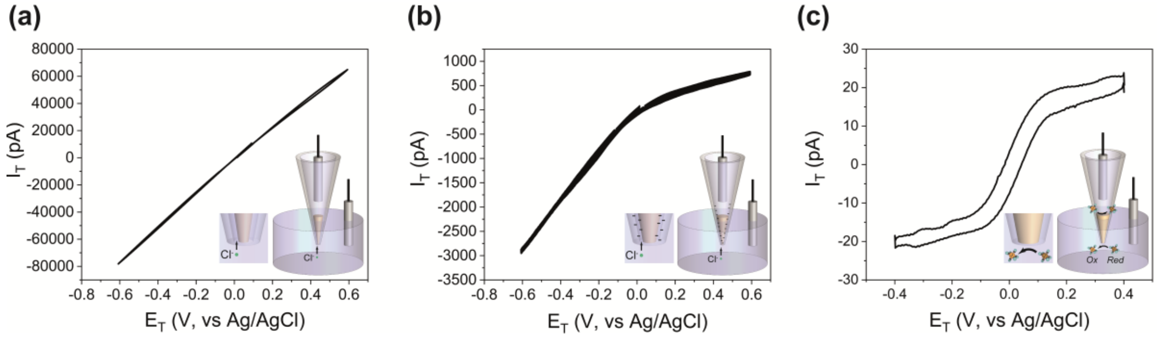

Figure S13. Different voltammetry responses observed for bipolar Au NEs in 2 mM $Fe(CN)_6^{4-/3-}$ in 0.125 M KCl_{aq} electrolyte solution: (a) Cyclic voltammograms for bipolar NEs with bigger gap (a), smaller gap (b), and no gap (c) at the gold/glass interface. It is to note that no gap was observed for all the three electrodes under SEM before EC testing.

Supplementary Information 6 – Electrochemical Sharpening of Long-taper W Micro-contacts

Drop-off fabrication of W wires is a standard electrochemical technique for sharpening into W micro- and nanowires.[18,19] Here, a tungsten microwire (25 µm diameter, Goodfellow, 99.9%) was first inserted into a thinned glass capillary (Marienfeld-Superior; 80 × 0.6 mm) to make it straight and fixed using Torr Seal epoxy. Using a standard one-step drop-off method (**Figure S14**), 3.5 mm of the W wire was vertically immersed into a 2 M KOH etchant solution, with a DC potential applied between the wire and a Cu loop cathode to drive oxidative dissolution into WO^{2-} anions (**Figure S15a,b**). At this condition, the highest etching rate occurs slightly below the meniscus at the air–electrolyte interface, where vortex formation and ion accumulation promote necking.[18] This thinning process continues until the bottom part drops off, resulting in a sharp wire tip, as reflected by a continuous current decrease followed by a sudden drop. Voltage-dependent studies (**Figure S14**) showed that lowering the applied potential extends the taper length but is inefficient (e.g., ~4 min at 0.3 V), making it impractical for reproducible long-taper W contacts. To address this, we developed a two-step static etching approach that enables fabrication of long-taper W contacts with controllable profiles and very high reproducibility (>90%) (**Figures S15–S16**). The first step (incomplete etching) thins a long length of the wire, with the process controlled by monitoring a continuous ~50% current decrease (**Figure S15b,d**). The second step (final cutting) is performed on the thinned section after slightly lifting the wire (e.g., 1 mm) and applying a lower potential (e.g., 0.2 V), leading to a sudden current drop. This step controls both taper length and tip radius (**Figure S15c,e**). The resulting W micro-contacts exhibit an ideal geometry (~5 µm width and ~700 µm taper length) and mechanical strength for insertion through the narrow glass body of the Au NEs while providing a soft, spring-loaded electrical contact to the long Au deposit. The entire process was monitored under an optical microscope, with >90% success rate (**Figure S15g**). Compared to previously reported methods relying on alternating-current/pulsed biasing[20,21] or dynamic etching[22], our two-step protocol is considerably simpler and more reproducible.

It should be noted that in cases where exceptionally large Au crystals are grown by our polyol-based approach, the W etching step can be omitted altogether. In such cases, commercially available W microwires (25 µm or smaller) are sufficient for direct contacting (**Figure S17**).

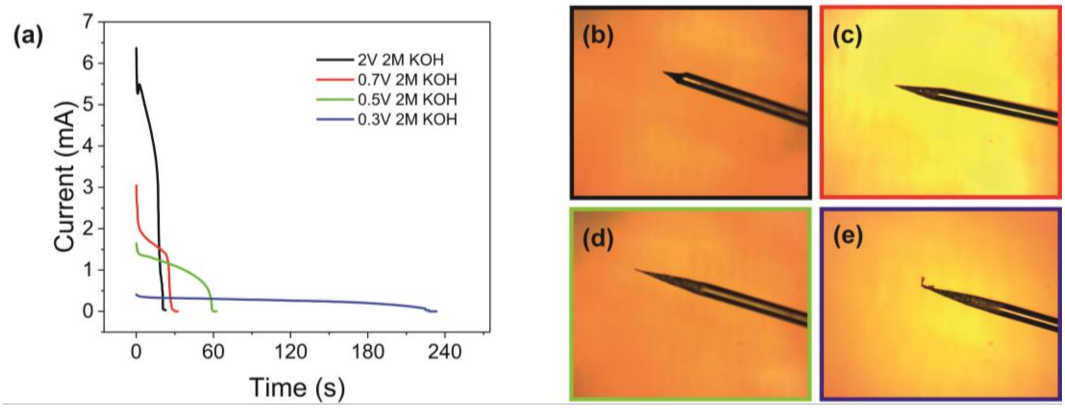

Figure S14. The effect of DC voltage in one-step electrochemical etching of W micro-wires. (a) Time trace of the current during the etching process at different applied voltages. Optical micrographs of the sharpened W wires fabricated at 2 V (b), 0.7 V (c), 0.5 V (d), and 0.3 V (e) applied potentials. A 2 M KOH etchant solution was used for all the experiments.

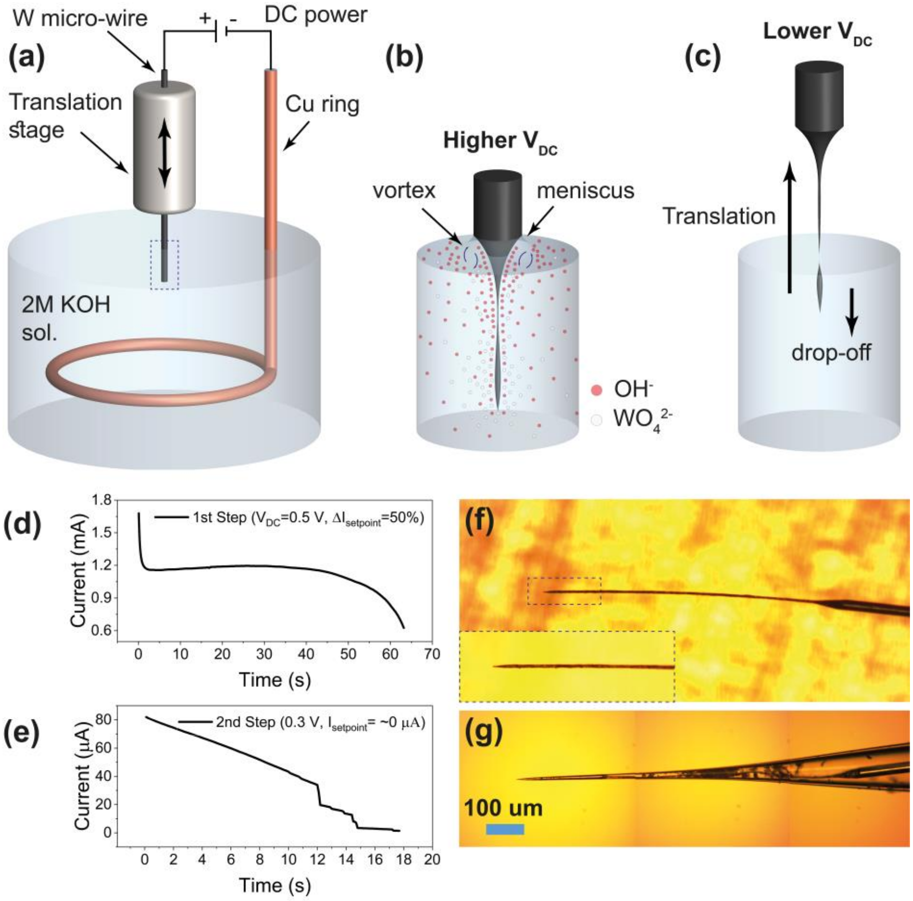

Figure S15. Two-step electrochemical etching of W micro-wires. (a) Schematic of the experimental setup consisting of a manual micro-stage for controlled positioning of the W micro-wire, a 10 ml beaker for the KOH etchant solution, and a potentiostat for applying a DC potential between the W wire and a Cu loop cathode. First-step incomplete etching (b) and chronoamperogram (d) of the W micro-wire at a relatively high DC voltage stopped after a 50% change in the current level. Second-step cutting (c) and chronoamperogram (e) of the thinned down W micro-wire at a lower applied voltage after an upward translation of the wire by the manual stage. (f) Optical micrograph of a typical electrochemically sharpened long-taper W micro-contact, and its use for physical contacting to the Au deposit under optical microscopy control.

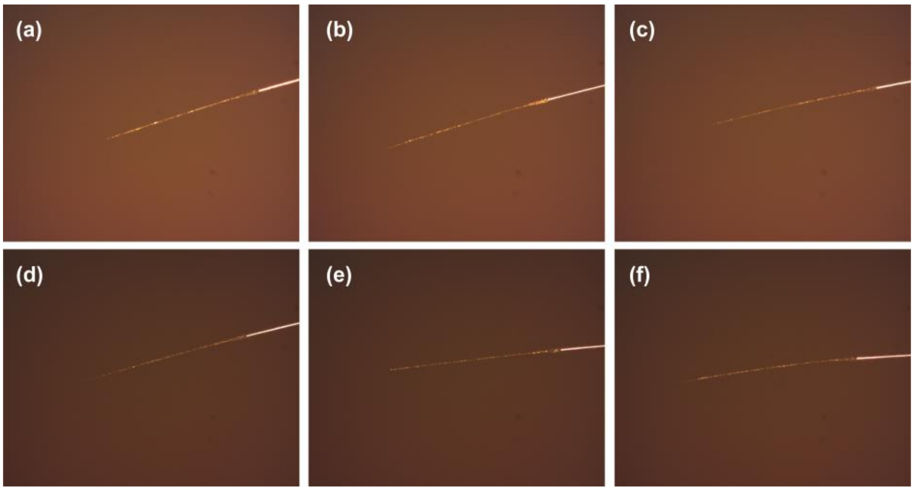

Figure S16. High reproducibility of the two-step approach for long-taper micro-contacts.

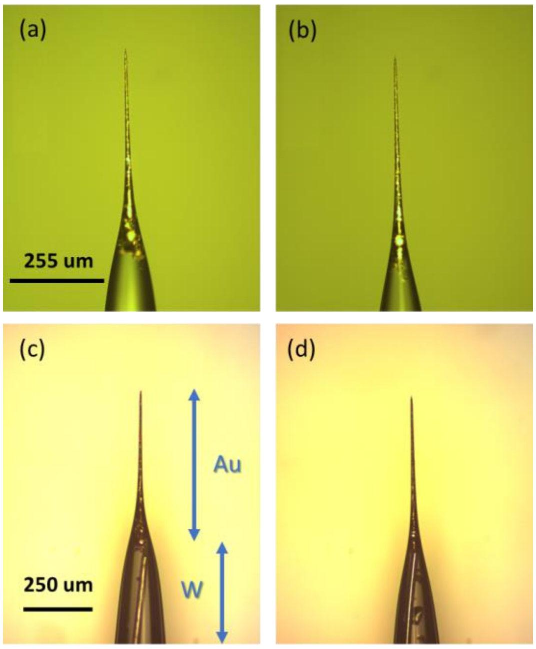

Figure S17. Facile physical contacting with commercial 25 μm-diameter W microwires. Selective growth of giant Au deposits with our chemical approach can enable a standard physical contacting with no need for electrochemical sharpening of W micro-wires.

Supplementary Information 7 – Material Characterization

Axial and cross-sectional FIB cuts and SEM imaging were used for precise characterization of the Au/glass interface and the growth mechanism of the Au deposit. Cross-sectional FIB cutting (see **Figure S21**) shows that there is no gap at the Au/glass interface and that the Au core clearly consists of multiple domains separated by straight boundaries, an indication of twin defects in face-centered cubic crystal structures.[23] A serial cross-sectional FIB cutting on the electrode shows both a displacement in the position of the twin boundaries and a change in their number. An axial FIB cut from the center of a Au NE (see **Figure 2h-j**) shows that the gap-free interface stays perfect even up to tens of micrometers away from the very end, and an axial twin boundary is observed across the length of the deposit, especially at upper parts.[24] SEM was unable to resolve the domain structure at the lower part of the electrode even with help of a low energy in-lens backscattered electron detector, nor to assess precisely the nature of the grain boundary defects.

Bright-field TEM (BF-TEM) imaging and crystal orientation mapping by precession-assisted TEM experiments were employed for further analysis of the structure of the NEs (see **Figure S19**). Experiments were performed on a small and a large-size electrode. TEM samples were prepared by making a cross-sectional lamella from the end of the NEs (see **Figure S18**). Briefly, the Au NEs were thoroughly coated with a ~10 nm carbon layer for decreasing the charging effects. Then, local carbon deposition was employed inside the FIB/SEM machine for handling a tiny cut of the Au NE by a sharp W manipulator and its transfer to a TEM grid. The samples were further thinned down to < 100 nm for TEM analysis. BF-TEM images of the electrodes showed a complete growth for both the Au NEs, with no gap at the Au/glass interface (see **Figure S19a,d**). Crystal orientation mapping from precession-assisted TEM experiment, using a Astar system[25], further revealed the orientation of the crystal domains with respect to the NE axis as well as the nature of the twin boundaries (see **Figure S19b,e**). Automatic indexation of the local pseudo-kinematic diffraction patterns collected in precession mode allows orientation mapping of the face-centered cubic Au crystal at nanoscale (see Methods, Transmission Electron Microscopy). The orientation map of the bigger electrode (**Figure S19b**) showed a close-to-

<111> bigger domain, i.e. [7, 5, 11], that is separated from two [-6,-4,-11]-oriented neighboring small domains by coherent {111} $\Sigma 3$ twin boundaries. Similarly, for the smaller electrode (**Figure S19e**), a close-to-<111> orientation, i.e. [7, 7, 11], was observed for the bigger domains, but with [-3,-3,-8] orientation for smaller domains, with again coherent {111} $\Sigma 3$ twin boundaries. As further evidences, the overlapping of the principal Bragg reflections together with a characteristic shift for other reflections in the selected area electron diffraction (SAED) (see **Figure S19c**) reveal the existence of crystal twinning[5]. The constant 60° misorientation angle in between the neighboring grains observed in the orientation maps (see **Figure S19f**) are clear evidences of the $\Sigma 3$ nature of the boundaries, characterized by a 60° rotation along a <111> crystal axis[23]. Moreover, the (111) pole figure analysis (see **Figure S20a,c**) reveal that for all crystal grains, (111) planes are aligned with the grain boundaries, proving their coherent nature (see the high-density points touching the edge of the pole figures in an orientation aligned with the grain boundary planes axes). The high intensity spots near [-1 1 1] orientation in inverse pole figures plot along NE axis (see **Figures S20b,d**) visually show the preferred close-to-<111> orientation for the studied electrodes, with a less misalignment for the smaller electrode. In numbers, the larger grain of the smaller NE has a <111> crystal axis oriented 12.7° away from the NE axis, whereas the larger grain of the bigger NE has a <111> crystal axis oriented 18.0° away from the NE axis. Although further studies are needed, the authors anticipate that even smaller nanoelectrodes (<100 nm radius) will exhibit orientations even closer to <111>. However, the small electrode size posed technical limitation for TEM lamella preparation by FIB cross-sectioning and were not observed in this study. This hypothesis is also in agreement with a change in growth trajectory within the conical nanopipette channel observed at micrometer scale during the growth process (see **Figure S22**) or with the twin boundaries displacement observed at nanometer scale by serial FIB cross-sectioning studies (see **Figure S21**), although this second observation can also be explained by the slightly misaligned <111> crystal axis with the pipette axis.

Overall, this single-crystalline (i.e. $\Sigma 3$ twinned) and gap-free nature for the chemically-grown Au NEs is unique as compared to nano-crystalline Au NEs fabricated by a laser sealing/pulling approach[26]. This unique crystallinity enhances electrochemical stability, surface reactivity and thus sensitivity for chemical detection, making these nanoelectrodes highly suitable for a wide range of practical applications (see **Supplementary Information 8**).

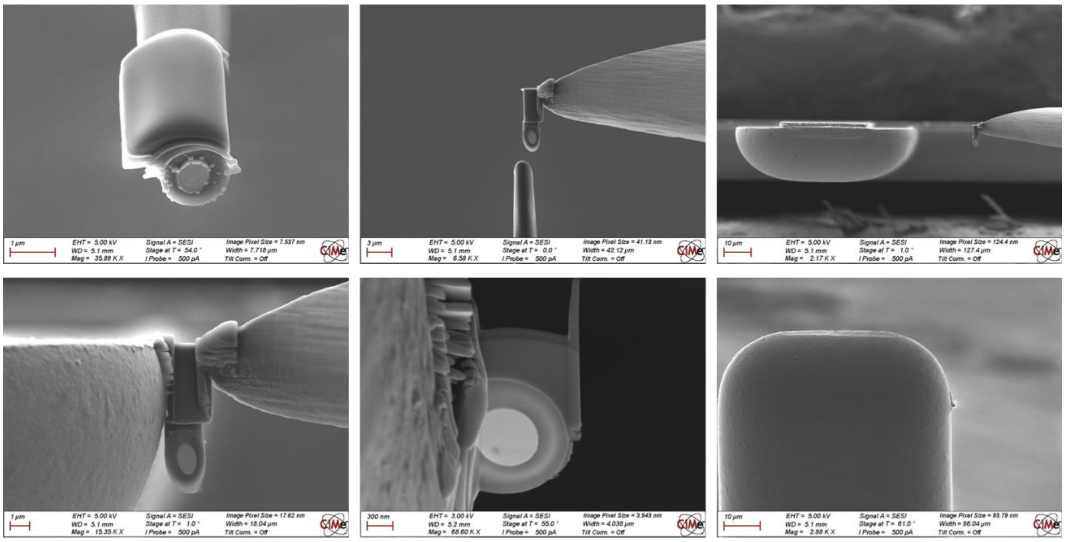

Figure S18. TEM lamella preparation from a cross-sectional slice of a Au NE tip.

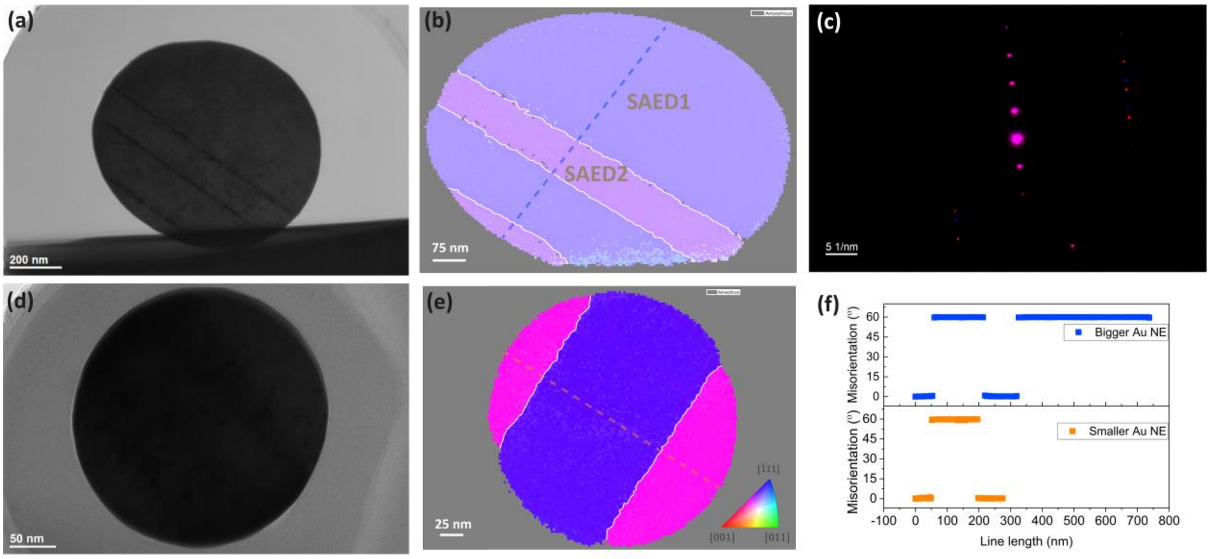

Figure S19. TEM analysis. BF-TEM image and crystal orientation map with respect to the NE axis (along z-direction) of a big (a,b) and a small (d,e) size Au NE. The colour legend for orientation maps in panels b and e is in inset. (c) Superimposed SAED patterns from positions 1 and 2 in panel b, respectively in red and blue. (f) Misorientation plots across the dashed lines shown in panels b and e.

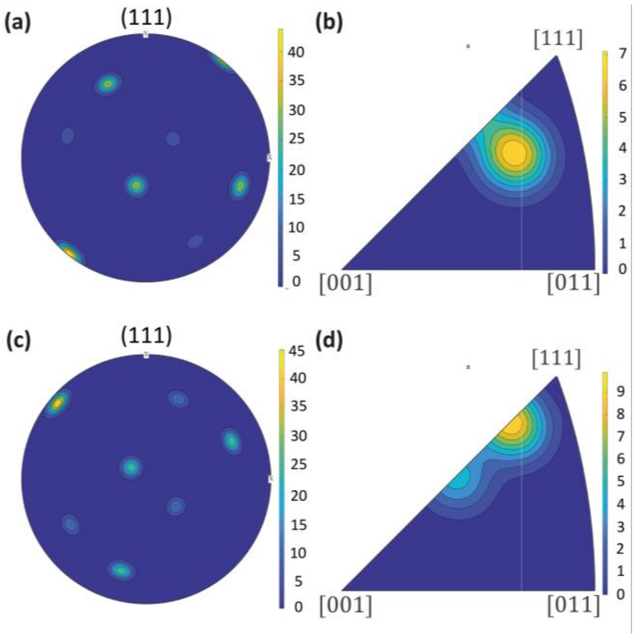

Figure S20. (111) pole figures and inverse pole figures along the nanoelectrode axis obtained from the crystal orientation maps of the studied gold nanoelectrodes (see **Figure S19b,e**). The (111) pole figures for the bigger (a) and smaller (c) electrodes show the distribution of (111) crystal planes orientations, and reveal the characteristic {111} Σ3 twin pattern. The inverse pole figures along the electrode axis for the bigger (b) and smaller (d) electrodes, where the high-intensity regions near the [111] highlight the preferred close-to-⟨111⟩ orientation of the crystal with respect to the electrode axis.

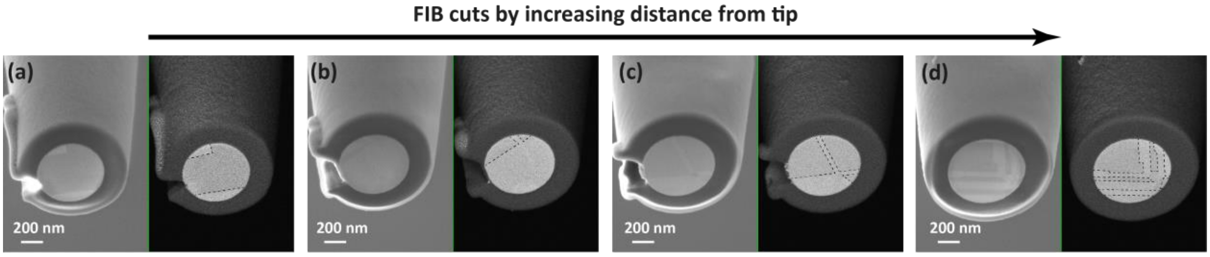

Figure S21. Serial FIB cross-sectioning on a single Au NE showing the evolution of the position of the {111} Σ3 twins along the axis of the NE.

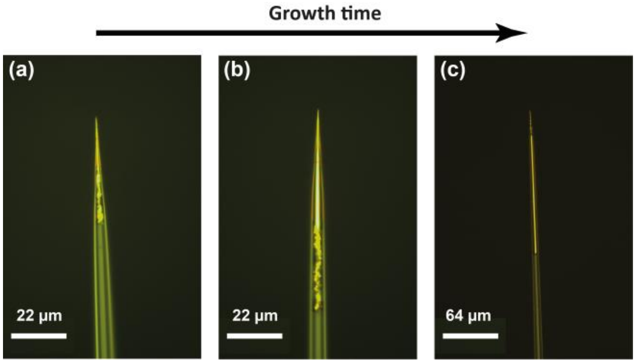

Figure S22. Reflection-mode optical images of a Au NE showing time evolution of the helical morphology of the growth front at different stages of the process.

Supplementary Information 8 – Electrochemical Characterization of Au electrodes

Electrochemistry, due to its unique capacity to probe the solid-electrolyte interface, is capable to detect imperfections beyond the resolution limit of advanced electron microscopy techniques.[27,28] However, despite their common use, the customary methods of electrochemical characterization via outer-sphere voltammetry, SECM approach curve, and roughness factor (RF) evaluation from blank CVs have serious limitations. In the following, we present exhaustive electrochemical data and discuss these limitations in relation to our nanoelectrodes characterization.

Limitations of Outer-sphere Electrochemical Measurements

Ideally, a perfect NE must show a low-hysteresis CV curve with sigmoidal-shape and clear mass diffusion limited plateau even at high scan rates. However, there are several reports that an imperfect NE/UME can also show a perfect outer-sphere CV[27,29] and vice versa. This is because outer-sphere reactions are not very sensitive to the surface composition and structure of the electrode.

We tested this method for our Au NEs characterization. **Figure S23** shows the SEM images and outer-sphere CV voltammograms of two gold nanoelectrodes, a well-sealed one (a,c) and a defective one (b,d), using both ferrocene methanol (FcMeOH; green curves) and ferrocyanide ($Fe(CN)_6^{4-}$; black and red curves) redox pairs. We observe that both the electrodes exhibit textbook-quality CVs when using FcMeOH, despite the presence of major sealing defects in the right electrode. Furthermore, we observe that both the electrodes exhibit a non-ideal CV (lack of diffusion-limited plateau, onset of hysteresis) when tested in $Fe(CN)_6^{4}$. This behavior indicates that the non-ideal CV does not arise from electrode imperfections but rather from an inner-sphere oxidative dissolution process between gold and cyanide, occurring concurrently with the outer-sphere electro-oxidation of $Fe(CN)_6^{4-}$.
Subsequently, we explored the role of the scan rate. **Figure S24** shows the CV curves of the same two electrodes in ferrocene methanol from 10 $mV.s^{-1}$ to $2V.s^{-1}$. We observe that even at very high scan rates, no significant difference between the two electrodes can be detected, despite their major difference in

sealing quality. Finally, we performed a stability test on these two electrodes (**Figure S26a-b**). Upon performing 50 cycles in FcMeOH, both nanoelectrodes exhibited stable response.

These results confirm prior literature observations that outer-sphere testing, while suitable to characterize the electrochemical surface area of the nanoelectrodes, cannot be reliably utilized to verify their structural integrity, in particular concerning the metal/glass sealing.

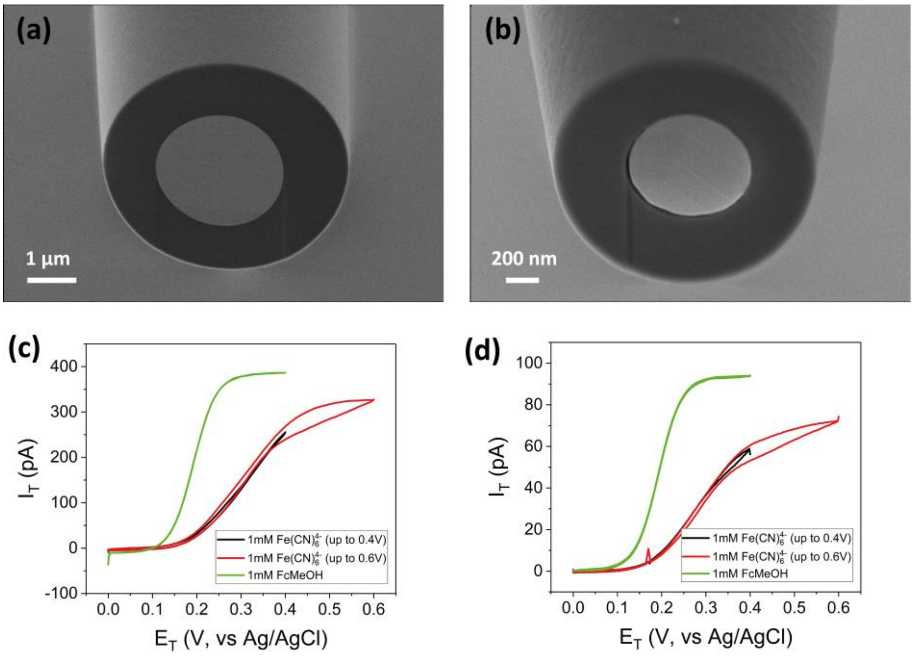

Figure S23. SEM images and cyclic voltammograms of two Au micro-/nanoelectrodes tested in 1mM ferrocene methanol (FcMeOH)/0.125M KCl and 1mM ferrocyanide $Fe(CN)_6^{4-}$/0.125M KCl electrolytes up to different oxidative potentials. Scan rate is 10 $mV.s^{-1}$.

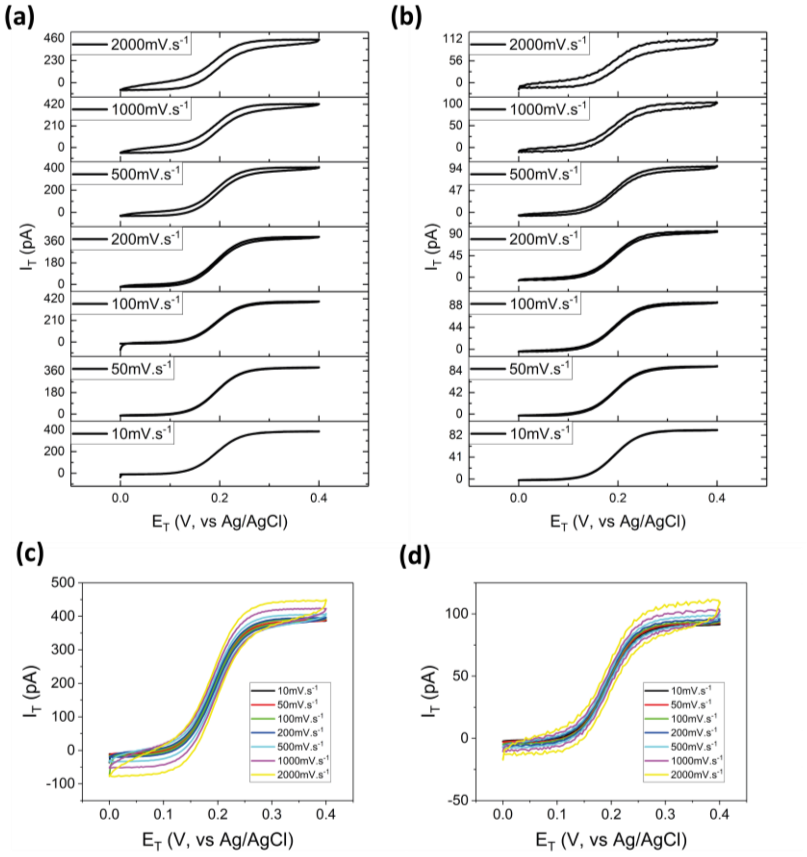

Figure S24. Cyclic voltammograms of the Au electrodes shown in **Figure S23a,b**. recorded in 1 mM FcMeOH/0.125M KCl at different scan rate from 10 mV.s^{-1} to 2V.s^{-1}. Panels c and d display the full sets of curves from panels a and b, respectively, combined in single plots.

Applicability and Limitations of Roughness Factor (RF) Calculations

Ideally, a perfect NE must show a RF value of between 1 and 2. However, evaluation of the RF value from calculation of the electrochemical and geometrical active surface areas is based on the assumption of only an oxidation and reduction of a 1:1 stoichiometric gold oxide/hydroxyl monolayer, which is extremely difficult to achieve practically at nanoscale.[30] Fast scan chronoamperometry and voltammetry experiments on Au UMEs have shown a scan rate dependency for the RF, with values up to 30 for a 0.1 V.s^{-1} scan rate, corresponding to a suppressed growth rate and different chemistry for the gold oxide formation after the monolayer formation.[31] Moreover, in situ ellipsometry[32], x-ray photoelectron spectroscopy[33], and electrochemical measurements[34] on single-crystalline Au (111) films have confirmed the formation of a multilayer gold oxide with thickness up to 1.2 nm and ~3 nm, which hugely depends on the previous potential history to the Au and sample preparation. Therefore, the reported RF values for NEs and UMEs obtained by blank CV are often overestimated, typically in the 10–12 range and up to 100, due to multilayers formation, resulting in an intrinsic uncertainty in quality evaluation of the perfect NEs by this technique. **Figure S25** shows the huge dependency of the integrated reduction charge increases to the maximum potential applied, resulting in inaccuracy in calculation of electrochemical surface area.

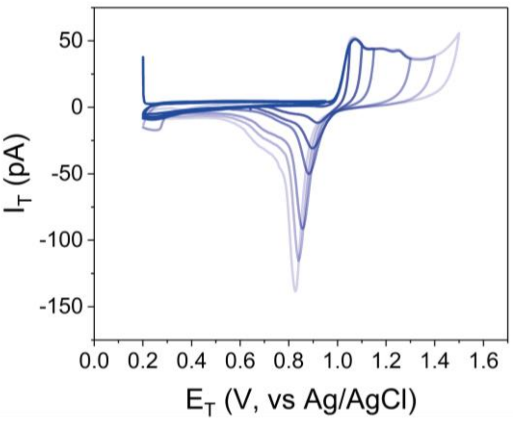

Figure S25. Cyclic voltammograms of a gold electrode in 50 mM H_2SO_4 electrolyte. The color contrast curves show different CVs performed up to different oxidative potentials. Scan rate is 10 mV.s^{-1}.

Inner-sphere Electrochemical Characterization

Recently, a precise electrochemical assessment method has been reported for analysis of the sealing quality for Pt disc UME tips.[35] This is based on analysis of the electrodes in the double-layer (DL) and hydrogen evolution reaction (HER) regimes, where there is no Faradic redox process and a possible surface diffusion/spillover of hydrogen at the Au/glass interface, respectively.

We tested the same two electrodes discussed above performing 50 CV cycles in H_2SO_4, across a potential window encompassing the HER and gold oxidation potentials (**Figure S26c-d**). Contrary to the case of outer-sphere testing (**Figure S26a-b**), these curves showed pronounced differences between the non-defective (left) and defective (right) electrodes. In particular, the Au nanoelectrode exhibits instability in the gold redox regime and significant current variations in the HER regime (panel d) due to structural imperfections. By contrast, the Au microelectrode, owing to its near-ideal geometry, remains stable and shows highly retraceable curves over all 50 cycles.

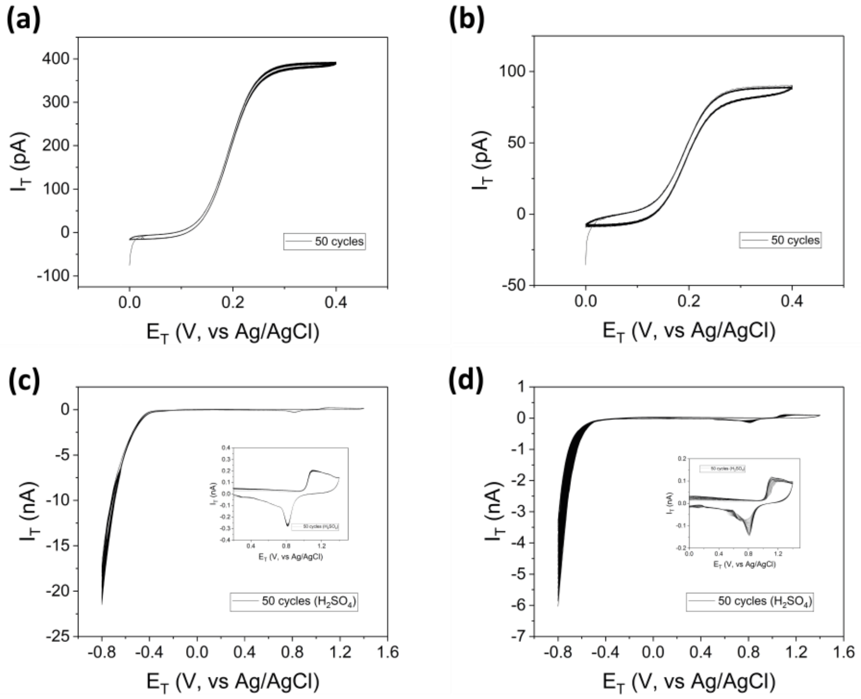

Figure S26. Outer- and Inner-sphere based stability analysis. Cyclic voltammograms (50 cycles) recorded in 1 mM FcMeOH/0.125 M KCl and 50 mM H_2SO_4 solutions for the microelectrode (a, c) and nanoelectrode (b, d) shown in **Figure S23a,b**, at scan rates of 100 mV·s^{-1} and 50 mV·s^{-1}, respectively.

Additionally, it is expected that the DL capacitance (C_{dl}) data and the tilt aspect of the CVs can reveal tiny gaps at the Au/glass interface even for electrodes that show perfect outer-sphere CVs. We implemented such a rapid assessment method for quality evaluation of our Au NEs. As it was not reported before for small-size Au NEs, we first evaluated the sensitivity of this method to sealing quality by performing experiments on selected defective NEs having different gap sizes. **Figure S27** and **S29a** show the quality assessment data for the defective Au NEs. Interestingly, we observed a tilted asymmetric CV in the DL regime specially at high scan rates, a more than 2 times increase in the C_{dl} value at scan rates smaller than 50 $mV.s^{-1}$, and an unreproducible CV within the HER-to-Au redox regime for the defective NEs, all increasing by the gap size.

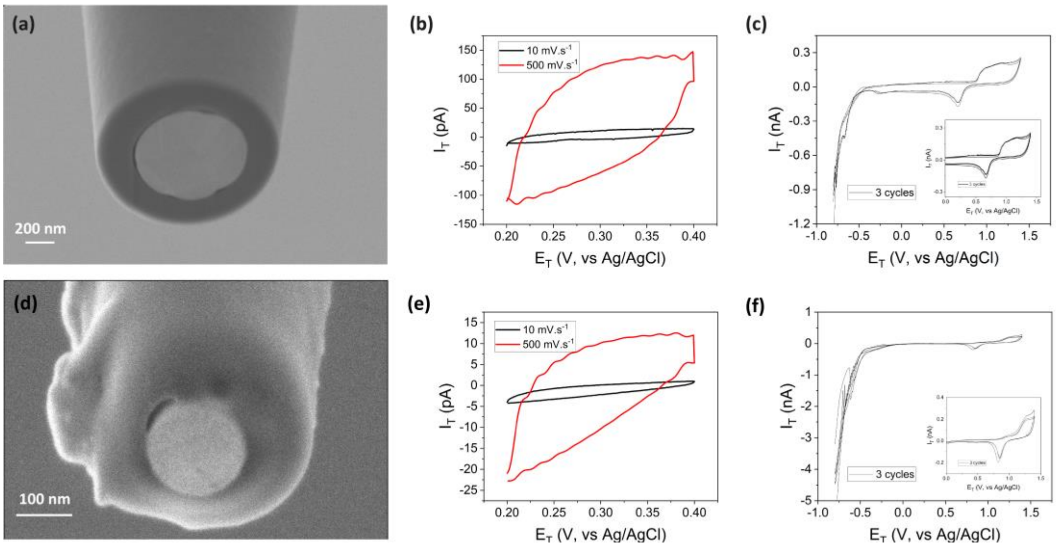

Figure S27. Electrochemical assessment of sealing quality for imperfect Au NEs having different core and gap sizes. SEM images (a,d) and cyclic voltammograms of the electrodes in the double-layer (b,e) and HER (c,f) regimes. Insets in panels c, f, and i show a zoomed-in view of three consecutive cycles of voltammograms within the gold redox regime. The experiments were performed in a 50 mM H_2SO_4 electrolyte solution at a 10 $mV.s^{-1}$ scan rate.

Overall, these control results confirm that inner-sphere characterization is a much more sensitive method to assess the gold nano/microelectrodes quality and we will therefore employ it in the rest of the work.

Size-dependent Electrochemical Performance of Au NEs

After these control tests, we employed these techniques for evaluation of our perfect looking Au NEs having different sizes.

Core size of thin-wall disc-shape electrodes can be estimated from the modified Sato's equation (eq.1) consisting of a *g factor* for consideration of the RG value of different size electrodes. **Table S3** lists g factors calculated for a few common RG values.

Table S3. Selected values of the parameter g with respect to RG in modified Saito's equation.

**RG**	∞	10	2	1.2
**g**	4	4.07	4.44	4.95

When performing inner-sphere analysis (see **Figure S28** and **29b**), all the tested Au NEs showed a flat CV response in the DL regime, a slight (< 2 times) scan rate dependency for the C_{dl}, and a very reproducible CV within the HER-to-Au redox regime. Interestingly, smaller size electrodes showed a positive shift for the Au reduction peak, a higher HER activity (i.e. current level at -0.8 V vs Ag/AgCl),

stronger H adsorption/desorption peaks, and higher C_{dl} values. We do not have a good explanation for the observed size dependency for Au redox peak and the HER activity, but in addition to some size effects, such as curvature and edge effects[36], we think it can be attributed to the observed different crystallographic orientations for different size electrode (see **Figure S19b,e** and **Figure S20b,d**); smaller electrodes which are closer to the closed-pack [111] orientation have higher number of atoms exposed which can enhance the H adsorption/desorption processes[37], and thus increase the C_{dl} value, resulting in higher activity for small size electrodes.[37,38] This can eventually promise a higher sensitivity and thus a higher signal-to-noise ratio for the small-size Au NEs for detection of chemical species, which is of utmost importance for practical application of NEs.

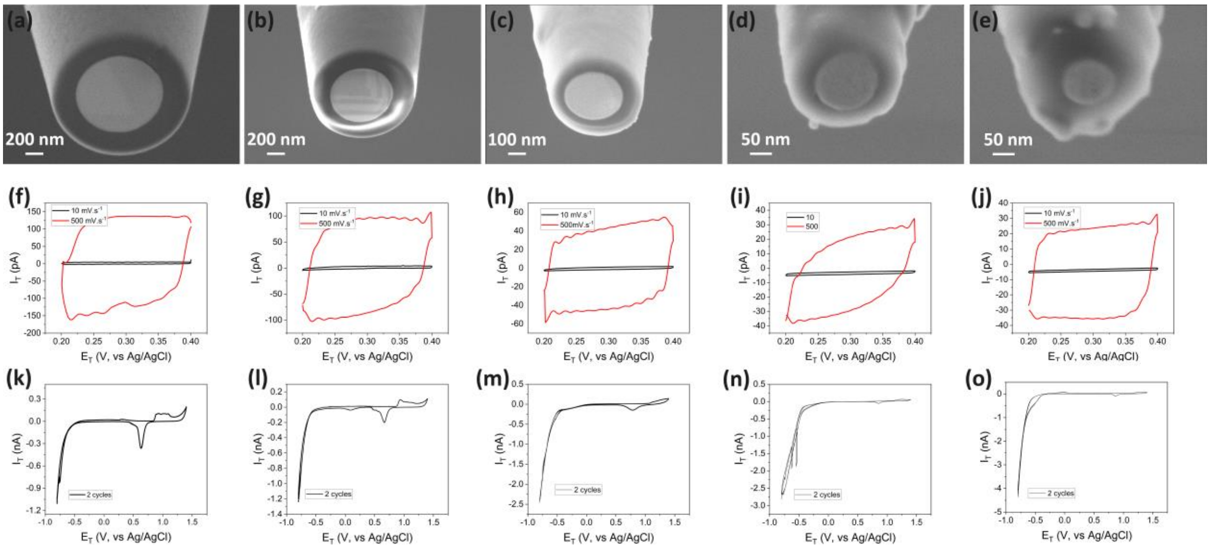

Figure S28. Electrochemical assessment of sealing quality for different core size Au NEs reported in **Figure S30** and **Figure 4g,h**. SEM images (a-e) and cyclic voltammograms of the electrodes in the double-layer (f-j) and HER (k-o) regimes. The HER experiments were performed in a 50 mM H_2SO_4 electrolyte solution at a 10 mV.s^{-1} scan rate.

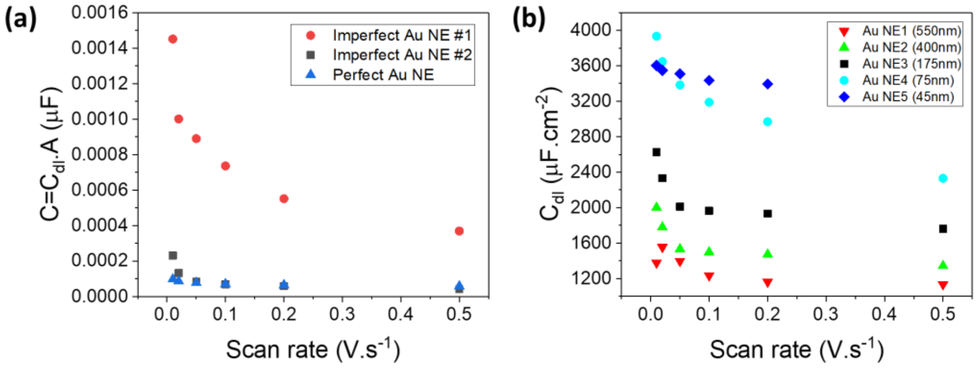

Figure S29. Quantitative analysis of scan rate dependent capacitance data for quality assessment of Au NEs. (a) Variation of total capacitance with scan rate for the perfect Au NE in **Figure 2d** and imperfect NEs in **Figure S27a,d**. (b) Plot of C_{dl} versus scan rate for the Au NEs in **Figure S30a-e**. The capacitance values were calculated from the CVs in the DL regime in a 50 mM H_2SO_4 electrolyte solution, and by considering the electrochemical surface area of the electrodes. Note that the variation in C_{dl} is < 2 times for all the NEs.

Outer-sphere reactions were instead used to verify the robust electrical connection and quantify the electrodes size.

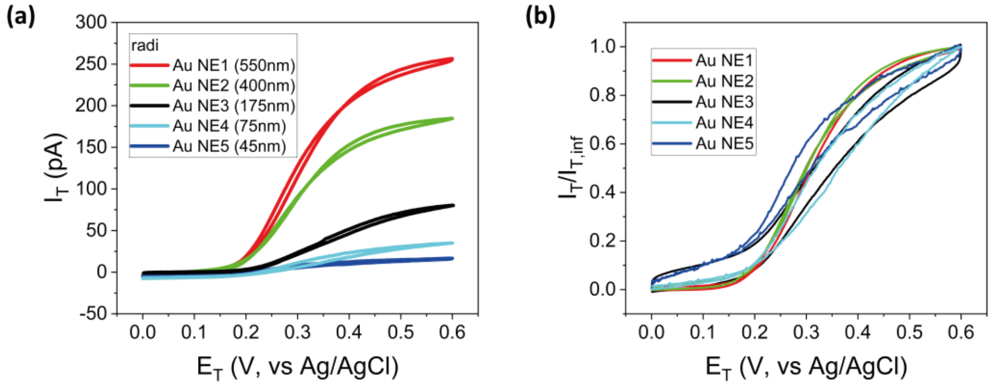

Figure S30. Outer-sphere testing results in ferrocyanide solution. (a) Cyclic voltammograms of disc-shape Au NEs/UMEs having different radii in an electrolyte solution containing 2 mM $Fe(CN)_6^{4-}$ in 0.25 M Na_2SO_4. (b) The same voltammograms normalized to their respective steady-state diffusion-limited current values, enabling direct comparison of electrode behavior independent of absolute current levels.

Reusability and long-term air stability

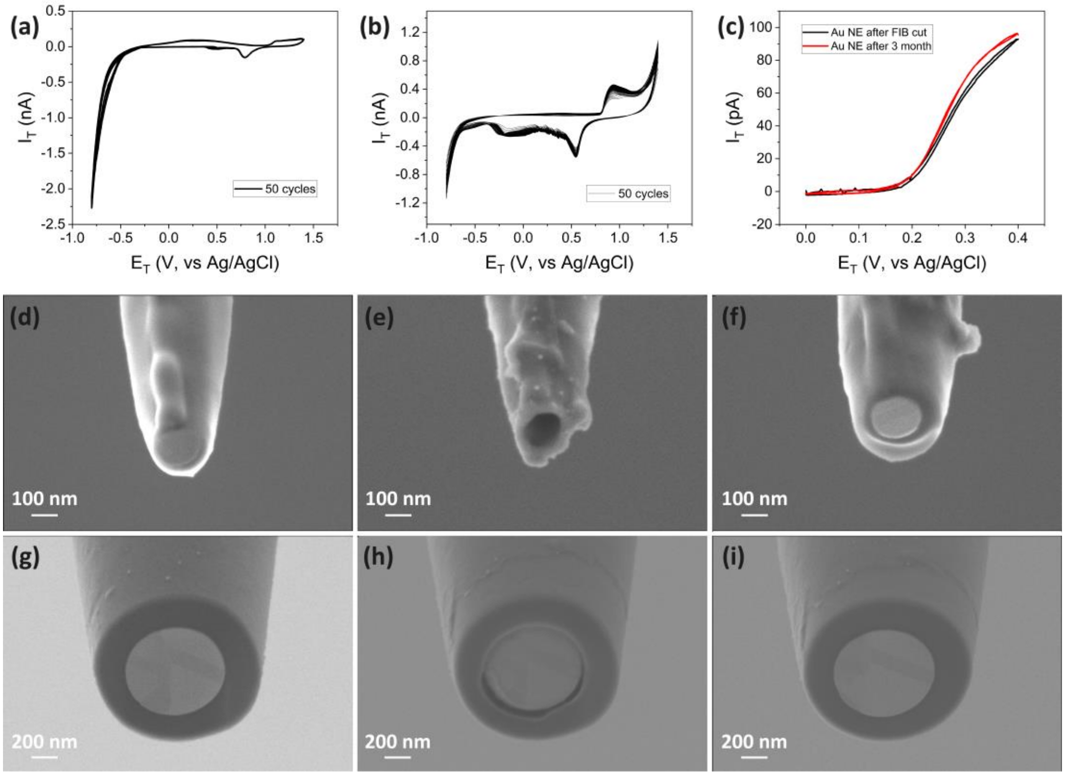

Figure S31. Electrochemical stability analysis of Au NEs. The recorded 50-cycles CVs in 50 mM H_2SO_4 solution at a 50 mV.s^{-1} scan rate for the Au NE in panel d (a) and panel g (b). (c) Cyclic voltammograms of the Au NE in panel f after FIB cutting (black curve) and after 3 months storage in air tested in a 2 mM $Fe(CN)_6^{4-}$ in 0.25 M $Na_2SO_{4,aq}$ solution. SEM images of the electrodes in before (d,g) and after (e,h) 50 cycles of CV in a 50 mM H_2SO_4 solution, as well as after a recovering FIB cutting process (f,i).

Photo-SECM Measurements

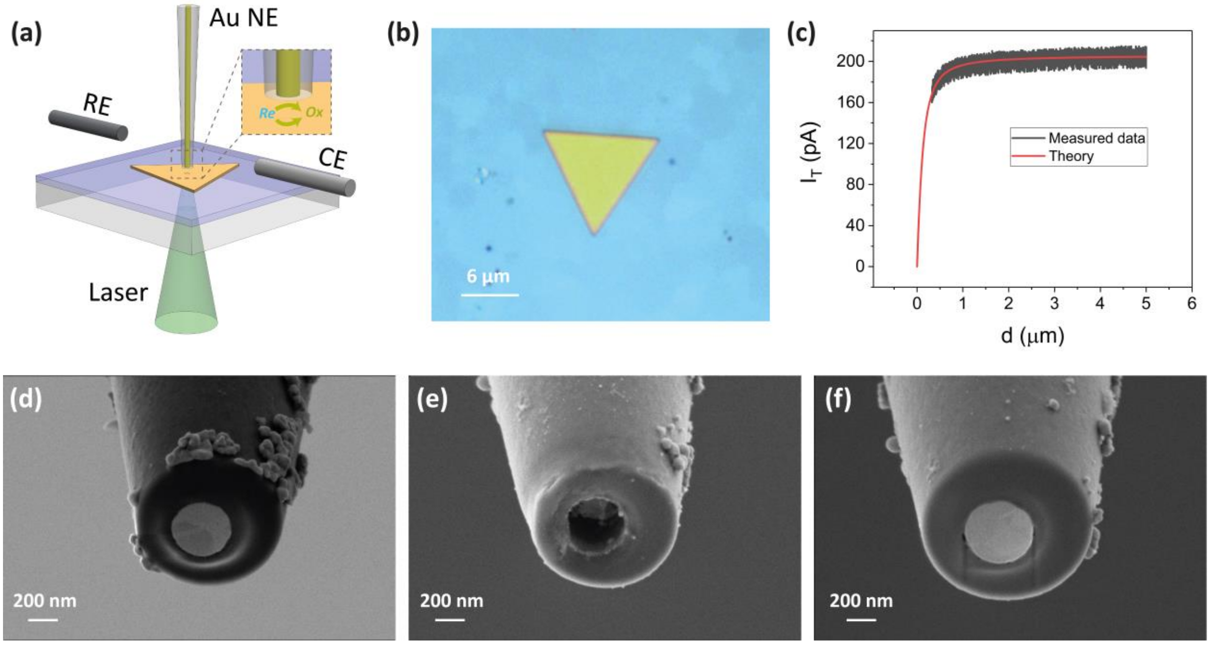

Figure S32. (a) Schematic of the gold micro-flake (Au MF) photocatalyst and photo-SECM configuration in competition experiment mode. A single-crystalline Au MF on TiO_2/ITO substrate is in contact with an electrolyte contacting 4 mM $Fe(CN)_6^{4-}$ (*Red*) redox molecule and 0.25 M Na_2SO_4. A gold NE is biased at 0.4 V vs Ag/AgCl (reference electrode, RE), and the substrate is at open circuit condition. Light is incident on the Au MF from the bottom. The same oxidation reaction occurs at the tip electrode and substrate surface. The current is measured through the tip electrode (working electrode, WE). A Pt wire is used as a counter electrode (CE) to complete the circuit. (b) Reflection-mode optical image of a typical two-dimensional Au MF catalyst structure on TiO_2/ITO substrate. (c) Approach curve recorded in the dark for positioning a 215 nm-radius Au NE at a 25% setpoint distance (~ 315 nm) prior to photo-SECM measurements, with a scan rate of 5 nm/s. Tilted-view SEM images of the Au NE in before (d) and after (e) ~7 hours of approach curve and photochemical imaging experiments in the redox solution using a 516 nm focused laser. (f) SEM image of the tip after a recovering FIB cutting process.

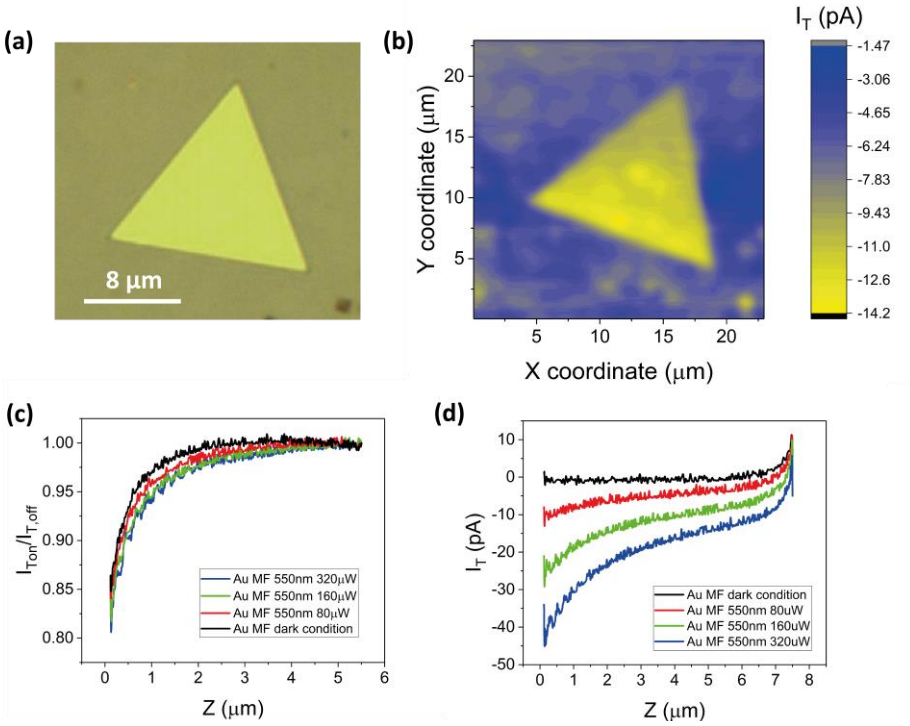

Figure S33. Optical micrograph of a Au microflake on TiO_2 substrate (a) and photo-SECM image (b) of photo-oxidation of $Fe(CN)_6^{4-}$ molecules on its surface. The image was obtained with a 350 nm-radius Au NE biased at a 0 V vs Ag/AgCl in a 4 mM $Fe(CN)_6^{4-}$ in 0.25 M Na_2SO_4 solution, and a 550 ± 10 nm focused laser excitation (80 µW). Panels (c) and (d) show approach curves recorded on Au microflake in dark and under a 550 ± 10 nm laser illumination (80-320 µW) in competition (E_T=0.4 V vs Ag/AgCl) and SG/TC (E_T=0 V vs Ag/AgCl) modes, respectively. Increasing laser power leads to more negative feedback in competition mode and more positive feedback in SG/TC mode, confirming the occurrence of photo-oxidation on the Au microflake surface.

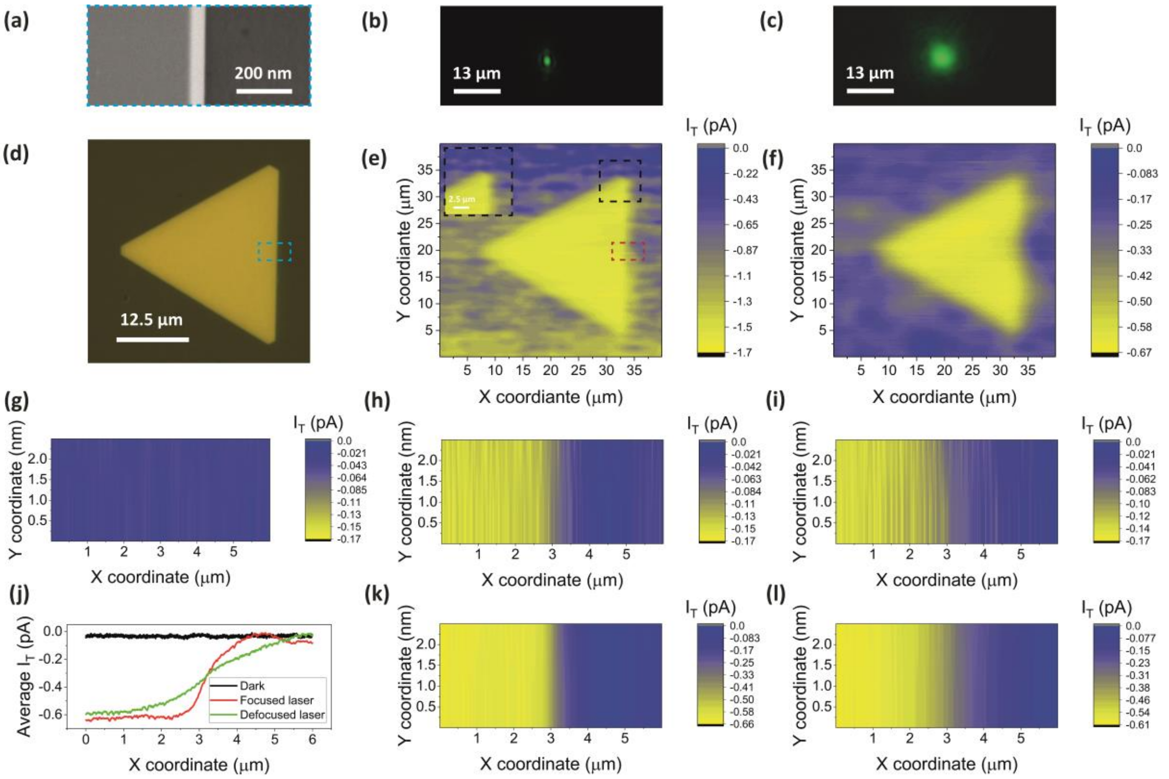

Figure S34. Effect of illumination conditions on photo-SECM spatial resolution. (a) Zoomed SEM image of the edge and (d) optical micrograph of the full geometry of a 40-nm-thick Au microflake on a TiO_2 substrate. (b, c) Optical micrographs of the focused (~1.3 µm FWHM) and defocused (~6.5 µm FWHM) 550 ± 10 nm laser beams used for photo-SECM imaging, respectively. (e, f) Constant-height continuous-scan photo-SECM images acquired using a 200 nm-radius Au NE tip in SG/TC mode (E_T= -0.2 V vs Ag/AgCl) with the focused (e) and defocused (f) 550 nm beams at an illumination power of 10 µW (256 pixels, scan speed of 16 µm.s^{-1}). The inset shows a magnified view of the flake corner indicated by the black dashed rectangle. Dark SECM image (g) and photo-SECM images of the flake edge shown in panel (a), indicated by the gray dashed rectangle in panel (e), acquired using the focused (h) and defocused (i) 550 nm laser beams at 1 µW illumination power. (k,l) Photo-SECM images of the flake edge obtained using the focused (k) and defocused (l) 550 ± 10 nm laser beams at 10 µW illumination power. (j) Average line profiles of the tip current across the flake edge under dark, focused-beam, and defocused-beam conditions at 10 µW illumination power. All the experiments were performed in a 4 mM $Fe(CN)_6^{4-}$ in 0.25 M Na_2SO_4 solution at the same tip-substrate distance.

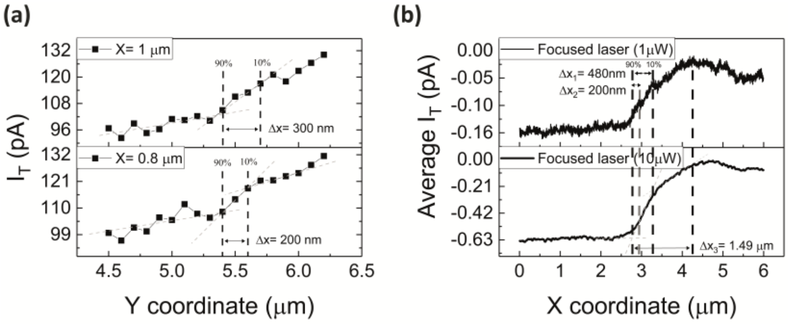

Figure S35. Line scans and 10–90% edge-width criterion for spatial-resolution estimation. (a) Line scans across the flake edge corresponding to Figure 4k (b) Average line scans across the flake edge corresponding to the photo-SECM images shown in **Figure S34h** and **Figure S34k**. Primary and secondary lateral resolutions are extracted based on the different slopes observed in the current transitions.

Limit-of-Detection (LOD) in bulk solution

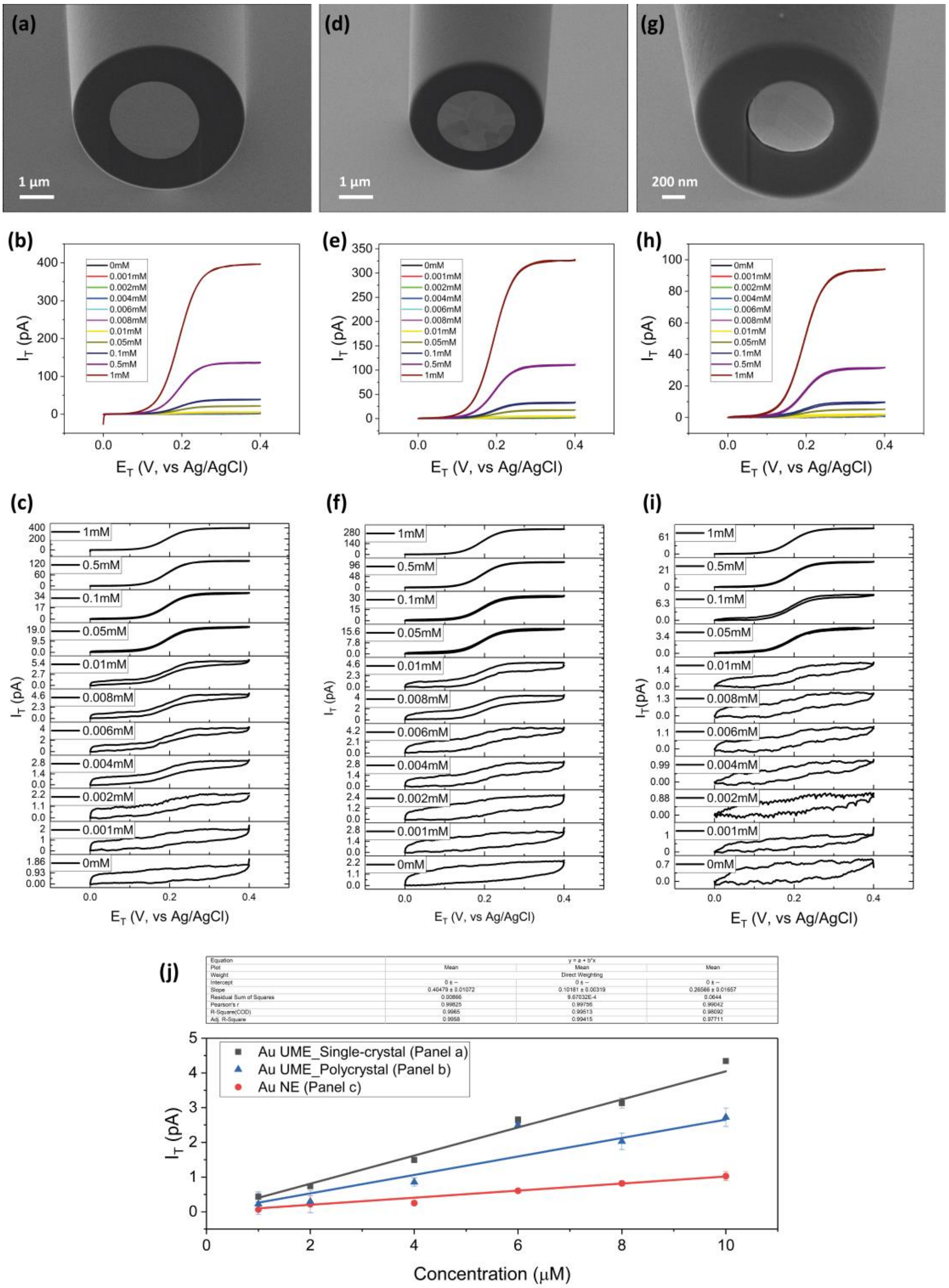

Figure S36. Detection sensitivity and limit of detection analysis. SEM images and cyclic voltammograms of a twinned single-crystalline Au UME (a-c), a polycrystalline Au UME (d-f), and a single-crystalline Au NE (g-i). (j) The $I_{T,Faradaic}$ vs concentration plot for the three electrodes, with slopes and standard deviations used to estimate detection sensitivity and limit of detection (LOD). Measurements were performed in FcMeOH/0.125M KCl electrolyte solutions at 10 $mV.s^{-1}$ inside a home-built faraday cage. $I_{T,Faradaic}$ was obtained by subtracting the capacitive charging current (measured in blank 0.125 M KCl) from the current difference between the oxidative and reductive threshold values. LOD values were calculated 79.4 nM, 187.1 nM, and 94.0 nM for the single-crystalline UME, polycrystalline UME, and single-crystalline Au NE, respectively.

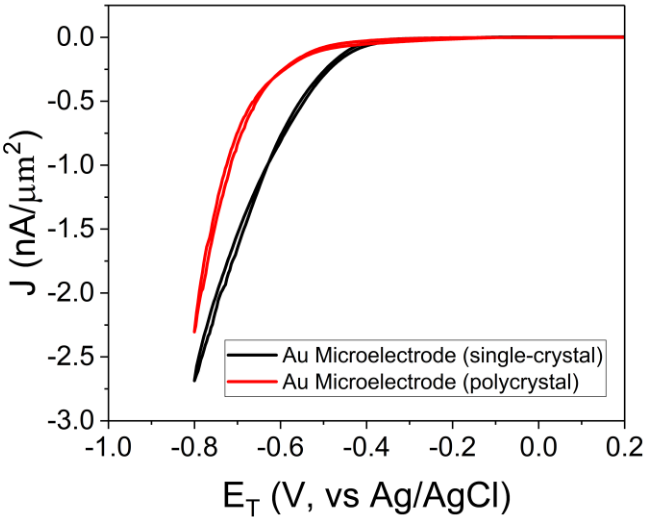

Figure S37. Cyclic voltammograms for hydrogen evolution on the twinned single-crystalline and polycrystalline Au UMEs reported in **Figure S36a,d** measured in 50 mM H_2SO_4 at 10 mV s^{-1} scan rate.

References

(1) *Scanning Electrochemical Microscopy*, 3rd ed.; Bard, A. J., Mirkin, M. V., Eds.; CRC Press: Boca Raton, 2022. https://doi.org/10.1201/9781003004592.

(2) Kiani, F.; Bowman, A. R.; Sabzehparvar, M.; Karaman, C. O.; Sundararaman, R.; Tagliabue, G. Transport and Interfacial Injection of D-Band Hot Holes Control Plasmonic Chemistry. *ACS Energy Lett.* **2023**, *8* (10), 4242–4250. https://doi.org/10.1021/acsenergylett.3c01505.

(3) Steinbock, L. J.; Steinbock, J. F.; Radenovic, A. Controllable Shrinking and Shaping of Glass Nanocapillaries under Electron Irradiation. *Nano Lett.* **2013**, *13* (4), 1717–1723. https://doi.org/10.1021/nl400304y.

(4) Sze, J. Y. Y.; Kumar, S.; Ivanov, A. P.; Oh, S.-H.; Edel, J. B. Fine Tuning of Nanopipettes Using Atomic Layer Deposition for Single Molecule Sensing. *Analyst* **2015**, *140* (14), 4828–4834. https://doi.org/10.1039/C5AN01001B.

(5) Kiani, F.; Tagliabue, G. High Aspect Ratio Au Microflakes via Gap-Assisted Synthesis. *Chem. Mater.* **2022**, *34* (3), 1278–1288. https://doi.org/10.1021/acs.chemmater.1c03908.

(6) Gao, R.; Lin, Y.; Ying, Y.-L.; Hu, Y.-X.; Xu, S.-W.; Ruan, L.-Q.; Yu, R.-J.; Li, Y.-J.; Li, H.-W.; Cui, L.-F.; Long, Y.-T. Wireless Nanopore Electrodes for Analysis of Single Entities. *Nat. Protoc.* **2019**, *14* (7), 2015–2035. https://doi.org/10.1038/s41596-019-0171-5.

(7) *High-Throughput Nanocapillary Filling Enabled by Microwave Radiation for Scanning Ion Conductance Microscopy Imaging | ACS Applied Nano Materials*. https://pubs.acs.org/doi/10.1021/acsanm.0c01345 (accessed 2025-01-22).

(8) Huang, W.; Gan, L.; Yang, H.; Zhou, N.; Wang, R.; Wu, W.; Li, H.; Ma, Y.; Zeng, H.; Zhai, T. Controlled Synthesis of Ultrathin 2D β-In2S3 with Broadband Photoresponse by Chemical Vapor Deposition. *Adv. Funct. Mater.* **2017**, *27* (36), 1702448. https://doi.org/10.1002/adfm.201702448.

(9) Singhal, R.; Bhattacharyya, S.; Orynbayeva, Z.; Vitol, E.; Friedman, G.; Gogotsi, Y. Small Diameter Carbon Nanopipettes. *Nanotechnology* **2009**, *21* (1), 015304. https://doi.org/10.1088/0957-4484/21/1/015304.

(10) *Carbon Nanopipette Electrodes for Dopamine Detection in Drosophila | Analytical Chemistry*. https://pubs.acs.org/doi/10.1021/ac504596y (accessed 2025-01-22).

(11) Gao, R.; Ying, Y.-L.; Li, Y.-J.; Hu, Y.-X.; Yu, R.-J.; Lin, Y.; Long, Y.-T. A 30 Nm Nanopore Electrode: Facile Fabrication and Direct Insights into the Intrinsic Feature of Single Nanoparticle Collisions. *Angew. Chem.* **2018**, *130* (4), 1023–1027. https://doi.org/10.1002/ange.201710201.

(12) Guerrette, J. P.; Oja, S. M.; Zhang, B. Coupled Electrochemical Reactions at Bipolar Microelectrodes and Nanoelectrodes. *Anal. Chem.* **2012**, *84* (3), 1609–1616. https://doi.org/10.1021/ac2028672.
(13) Wood, M.; Zhang, B. Bipolar Electrochemical Method for Dynamic In Situ Control of Single Metal Nanowire Growth. *ACS Nano* **2015**, *9* (3), 2454–2464. https://doi.org/10.1021/acsnano.5b00139.
(14) *Gold etching for microfabrication | Gold Bulletin*. https://link.springer.com/article/10.1007/s13404-014-0143-z (accessed 2025-01-22).
(15) *Revisiting a classical redox process on a gold electrode by operando ToF-SIMS: where does the gold go? - Chemical Science (RSC Publishing)*. https://pubs.rsc.org/en/content/articlelanding/2019/sc/c9sc00956f (accessed 2025-01-22).
(16) Hao, R.; Zhang, B. Nanopipette-Based Electroplated Nanoelectrodes. *Anal. Chem.* **2016**, *88* (1), 614–620. https://doi.org/10.1021/acs.analchem.5b03548.
(17) Zhu, X.; Qiao, Y.; Zhang, X.; Zhang, S.; Yin, X.; Gu, J.; Chen, Y.; Zhu, Z.; Li, M.; Shao, Y. Fabrication of Metal Nanoelectrodes by Interfacial Reactions. *Anal. Chem.* **2014**, *86* (14), 7001–7008. https://doi.org/10.1021/ac501119z.
(18) Chang, W.-T.; Hwang, I.-S.; Chang, M.-T.; Lin, C.-Y.; Hsu, W.-H.; Hou, J.-L. Method of Electrochemical Etching of Tungsten Tips with Controllable Profiles. *Rev. Sci. Instrum.* **2012**, *83* (8), 083704. https://doi.org/10.1063/1.4745394.
(19) Ju, B.-F.; Chen, Y.-L.; Ge, Y. The Art of Electrochemical Etching for Preparing Tungsten Probes with Controllable Tip Profile and Characteristic Parameters. *Rev. Sci. Instrum.* **2011**, *82* (1), 013707. https://doi.org/10.1063/1.3529880.
(20) Toh, S. L.; Tan, H.; Lam, J. C.; Hsia, L. C.; Mai, Z. H. Optimization of AC Electrochemical Etching for Fabricating Tungsten Nanotips with Controlled Tip Profile. *J. Electrochem. Soc.* **2009**, *157* (1), E6. https://doi.org/10.1149/1.3258289.
(21) Chang, W.-T.; Hwang, I.-S.; Chang, M.-T.; Lin, C.-Y.; Hsu, W.-H.; Hou, J.-L. Method of Electrochemical Etching of Tungsten Tips with Controllable Profiles. *Rev. Sci. Instrum.* **2012**, *83* (8), 083704. https://doi.org/10.1063/1.4745394.
(22) Khan, Y.; Al-Falih, H.; Zhang, Y.; Ng, T. K.; Ooi, B. S. Two-Step Controllable Electrochemical Etching of Tungsten Scanning Probe Microscopy Tips. *Rev. Sci. Instrum.* **2012**, *83* (6), 063708. https://doi.org/10.1063/1.4730045.
(23) Porter, D. A.; Easterling, K. E.; Sherif, M. Y. *Phase Transformations in Metals and Alloys*, 4th ed.; CRC Press: Boca Raton, 2021. https://doi.org/10.1201/9781003011804.
(24) *Atomic-Scale Mechanism of Unidirectional Oxide Growth - Sun - 2020 - Advanced Functional Materials - Wiley Online Library*. https://advanced.onlinelibrary.wiley.com/doi/10.1002/adfm.201906504 (accessed 2025-01-22).
(25) Viladot, D.; Véron, M.; Gemmi, M.; Peiró, F.; Portillo, J.; Estradé, S.; Mendoza, J.; Llorca-Isern, N.; Nicolopoulos, S. Orientation and Phase Mapping in the Transmission Electron Microscope Using Precession-Assisted Diffraction Spot Recognition: State-of-the-Art Results. *J. Microsc.* **2013**, *252* (1), 23–34. https://doi.org/10.1111/jmi.12065.
(26) J. Percival, S.; E. Vartanian, N.; Zhang, B. Laser-Pulled Ultralong Platinum and Gold Nanowires. *RSC Adv.* **2014**, *4* (21), 10491–10498. https://doi.org/10.1039/C3RA47207H.
(27) *Adsorption/Desorption of Hydrogen on Pt Nanoelectrodes: Evidence of Surface Diffusion and Spillover | Journal of the American Chemical Society*. https://pubs.acs.org/doi/full/10.1021/ja902876v (accessed 2025-01-22).
(28) Jacobse, L.; Raaijman, S. J.; Koper, M. T. M. The Reactivity of Platinum Microelectrodes. *Phys. Chem. Chem. Phys.* **2016**, *18* (41), 28451–28457. https://doi.org/10.1039/C6CP05361K.
(29) Nioradze, N.; Chen, R.; Kim, J.; Shen, M.; Santhosh, P.; Amemiya, S. Origins of Nanoscale Damage to Glass-Sealed Platinum Electrodes with Submicrometer and Nanometer Size. *Anal. Chem.* **2013**, *85* (13), 6198–6202. https://doi.org/10.1021/ac401316n.
(30) *Electrochemical Methods: Fundamentals and Applications, 3rd Edition | Wiley*. Wiley.com. https://www.wiley.com/en-ae/Electrochemical+Methods%3A+Fundamentals+and+Applications%2C+3rd+Edition-p-9781119334057 (accessed 2025-01-16).

(31) Zuliani, C.; Walsh, D. A.; Keyes, T. E.; Forster, R. J. Formation and Growth of Oxide Layers at Platinum and Gold Nano- and Microelectrodes. *Anal. Chem.* **2010**, *82* (17), 7135–7140. https://doi.org/10.1021/ac101728a.

(32) Pfeiffer, M.; Wu, X.; Ebrahimi, F.; Mameka, N.; Eich, M.; Petrov, A. Chemical Interface Damping by Electrochemical Gold Oxidation. *J. Phys. Chem. C* **2024**, *128* (37), 15686–15693. https://doi.org/10.1021/acs.jpcc.4c04586.

(33) Cook, K. M.; Ferguson, G. S. Determination of the Wavelength-Dependent Refractive Index of a Gold-Oxide Thin Film. *J. Phys. Chem. C* **2011**, *115* (46), 22976–22980. https://doi.org/10.1021/jp207031s.

(34) Valtiner, M.; Banquy, X.; Kristiansen, K.; Greene, G. W.; Israelachvili, J. N. The Electrochemical Surface Forces Apparatus: The Effect of Surface Roughness, Electrostatic Surface Potentials, and Anodic Oxide Growth on Interaction Forces, and Friction between Dissimilar Surfaces in Aqueous Solutions. *Langmuir* **2012**, *28* (36), 13080–13093. https://doi.org/10.1021/la3018216.

(35) Bodappa, N. Rapid Assessment of Platinum Disk Ultramicroelectrodes' Sealing Quality by a Cyclic Voltammetry Approach. *Anal. Methods* **2020**, *12* (27), 3545–3550. https://doi.org/10.1039/D0AY00649A.

(36) Wang, H.; Pilon, L. Accurate Simulations of Electric Double Layer Capacitance of Ultramicroelectrodes. *J. Phys. Chem. C* **2011**, *115* (33), 16711–16719. https://doi.org/10.1021/jp204498e.

(37) Perez, J.; Gonzalez, E. R.; Villullas, H. M. Hydrogen Evolution Reaction on Gold Single-Crystal Electrodes in Acid Solutions. *J. Phys. Chem. B* **1998**, *102* (52), 10931–10935. https://doi.org/10.1021/jp9831987.

(38) Marković, N. M.; Gasteiger, H. A.; Ross, P. N. Oxygen Reduction on Platinum Low-Index Single-Crystal Surfaces in Alkaline Solution: Rotating Ring DiskPt(Hkl) Studies. *J. Phys. Chem.* **1996**, *100* (16), 6715–6721. https://doi.org/10.1021/jp9533382.
